# Precursor- and waste-free synthesis of spark-ablated nanoparticles with enhanced photocatalytic activity and stability towards airborne organic pollutant degradation†

**DOI:** 10.1039/d3en00348e

**Published:** 2024-01-03

**Authors:** Sarka Drdova, Min Gao, Olga Sambalova, Robin Pauer, Zhouping Zhou, Sofia Dimitriadou, Andreas Schmidt-Ott, Jing Wang

**Affiliations:** a Institute of Environmental Engineering, ETH Zurich 8093 Zürich Switzerland jing.wang@ifu.baug.ethz.ch; b Laboratory for Advanced Analytical Technologies, Empa – Swiss Federal Laboratories for Materials Science and Technology 8600 Dübendorf Switzerland; c Electron Microscopy Center, Empa – Swiss Federal Laboratories for Materials Science and Technology 8600 Dübendorf Switzerland; d Chemical Engineering Department, Delft University of Technology 2600 AA Delft The Netherlands; e VSPARTICLE B.V 2629 JD Delft The Netherlands

## Abstract

Photocatalyst synthesis typically involves multiple steps, expensive precursors, and solvents. In contrast, spark ablation offers a simple process of electrical discharges in a gap between two electrodes made from a desirable material. This enables a precursor- and waste-free generation of pure metal oxide nanoparticles or mixtures of various compositions. This study presents a two-step method for the production of photocatalytic filters with deposited airborne MnO_*x*_, TiO_2_, and ZnO nanoparticles using spark ablation and calcination processes. The resulting MnO_*x*_ and TiO_2_ filters demonstrated almost twice the activity with outstanding performance stability, as compared to sol–gel MnO_2_ and commercial TiO_2_. The introduced method is not only simple, precursor- and waste-free, and leads to superior performance for the case studied, but it also has future potential due to its versatility. It can easily produce mixed and doped materials with further improved properties, making it an interesting avenue for future research.

## Abstract 2

Environmentally friendly and waste-free production of photocatalytic filters involving spark ablation and calcination processes for enhanced and durable photocatalytic performance.

## Graphical abstract

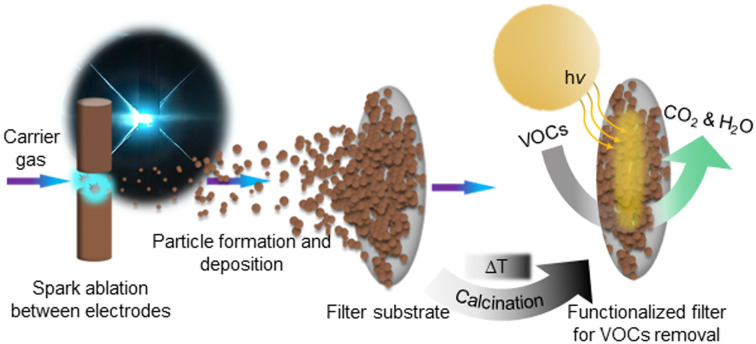

**Environmental significance**

The presented spark ablation and direct deposition of photocatalytic nanoparticles for the production of functionalized filters hold significant environmental and nanotechnology implications, offering a promising solution to address challenges associated with air pollution. Firstly, this innovative method reduces the environmental impact of photocatalyst production by eliminating complexity and the need for costly and potentially harmful precursors, while minimizing waste generation during the synthesis process. Secondly, this study highlights the importance of nanotechnology in advancing environmental sustainability, as the spark-ablated and calcined nanoparticles exhibited superior photocatalytic performance and durability compared to traditionally synthesized materials. The simplicity, sustainability, and adaptability to produce various materials and mixtures make the presented method a promising avenue for future research in environmental pollution remediation.

## Introduction

Atmospheric and indoor air pollutants, such as nitrogen and carbon oxides (NO_*x*_ and CO) and volatile organic compounds (VOCs), represent a serious risk for human health in indoor and outdoor environments. VOCs are widely used in both domestic and industrial activities.^1–3^ Many VOC emissions are toxic, carcinogenic, mutagenic, or teratogenic, and they can affect the building occupants' comfort and health, a phenomenon referred to as the sick building syndrome.^4^ VOC removal by adsorption is well established in environmental and indoor air control technologies. In this process, the pollutants are simply transferred from the gas phase onto the surface of the solid phase requiring additional treatment steps. On the other hand, photocatalysis based on semiconductors has proved to be an effective, inexpensive, and environmentally friendly process that enables the oxidation of VOCs into water and carbon dioxide. The traditional process of photocatalysis generates highly energetic electrons (e^−^) and holes (h^+^) upon light irradiation, which contribute to the formation of reactive oxygen species, such as O_2_˙^−^ and OH˙ radicals, capable of decomposing organic pollutants.^2^ Titanium dioxide (TiO_2_) and zinc oxide (ZnO) are by far the most commonly used and studied photocatalytic semiconductors mainly due to their exceptional optical and electronic properties, high efficiency, environmental harmlessness and low cost.^1^ Owing to their wide band gap, the conventional photocatalysts require UV irradiation for the activation, which makes them inapt for solar irradiation offering only about 4% of the UV spectrum. In order to utilize the full solar spectrum, visible-infrared light-driven catalysts have been extensively investigated mainly by catalyst doping, which enables reduction of the wide band gap.^5^ Recently, manganese oxide (MnO_2_) and its nanocomposites have been investigated for photocatalytic and photothermocatalytic performance owing to their effective activation by visible and infrared irradiation, which represent the majority of the solar spectrum.^6–9^ In addition to that, MnO_2_ is also attractive due to its inexpensiveness, earth-abundance, and environmental benignity.^8^ Similarly, Mn_3_O_4_, Mn_2_O_3_, and MnO have been investigated for their photocatalytic properties.^7,8,10^ Given the complexity of the driving mechanism that further exacerbated by the multitude of manganese oxide variations, the investigation of the primary driving mechanism in most cases poses a substantial challenge yet holds profound significance.^11^ It is believed that the oxidation activity is based on either light-driven thermal catalysis or the synergy of photochemical and thermochemical effects involving both photogenerated electron–hole pairs and the activation of surface lattice oxygen due to the temperature elevation.^6,12,13^

Although much effort has been devoted to the development of an efficient UV and solar-light-driven photocatalyst and understanding of the underlying mechanisms, there are several common challenges for practical applications, such as complex synthesis routes, various optical absorption properties, uncertain charge separation and transfer, and problematic photocatalytic stability.^2,5^ In general, the catalytic properties depend on the electronic structure of the surface of the catalyst that induces the reaction of adsorbed molecules. The surface properties and curvature affect the adsorption strength as well as the coordination number of catalyst surface atoms, which influences the number of dangling bonds at the surface and alters the electronic orbital configuration. This implies that photoactivity is strongly affected by the size and phase of a semiconductor material.^14,15^ We will show below that such properties can be optimized through a flexible nanoparticle synthesis method.

Traditionally, the production of semiconductor nanoparticles is performed by wet-chemistry routes, such as sol–gel and hydrothermal methods.^16^ These techniques provide unique possibilities for tailoring the size and phase of nanoparticles, but they employ precursor solutions that can lead to impurities and the production of hazardous waste. There are several gas-phase alternatives for nanoparticle production, such as a flame synthesis^17^ or material ablation using lasers and sparks.^18,19^ Both ablation-based and flame-based nanoparticle syntheses have their own merits and limitations and the choice between the two methods depends on specific requirements. In general, the main advantage of flame synthesis is the relatively high production rate whereas contamination during high-temperature reactions, limited material compatibility, and proper control of the synthesis parameters involving solvents and precursors are the main limitations.^17^ In contrast, laser ablation^18^ and spark ablation^19^ provide simple and easy-to-control, versatile, and environmentally friendly alternatives. The nanoparticle formation in these processes is based on the direct and local evaporation of bulk materials without the involvement of any precursors or waste production. Therefore, the ablation-based methods offer material versatility and high purity of produced nanoparticles.^20^ While these gas-phase methods offer a promising alternative for nanoparticle production, it is worth noting that a low production rate is the main limitation.^21^

Spark ablation production of catalytic nanoparticles has gained attention in recent years owing to its unique capability to generate pure metal or semiconductor nanoparticles, mixtures of various compositions,^22–24^ or their oxides. It is a waste-free synthesis that requires no expensive chemicals or precursors except the substance or mixture of substances intended to form the resulting nanoparticles. During the synthesis of spark ablated nanoparticles, short electrical discharges are induced in the gap between the two electrodes that consist of the substance or composite of choice. The sparks are generated in high frequency forming a plasma where the temperature can exceed 20 000 K. As a result, the electrodes are ablated by evaporation in a few microseconds. After extinction of the spark, the formed vapour is cooled by adiabatic expansion and by the surrounding stream of inert gas and it condenses to form atomic clusters and sub-20 nm nanoparticles, which are free from surfactants and precursors. The main advantages of spark ablation are i) simplicity and scalability of the process, ii) high purity of produced nanoparticles, iii) facile control of nanoparticle size in the size range where the electronic and optical properties and surface-to-volume characteristics extend bulk material properties significantly, iv) facile control of nanoparticle composition, and v) waste-free environment-friendly process.^15,25,26^ These characteristics have made this method a perfect candidate for various applications. In particular, the method has been used for the production of diverse materials such as pure metals (W, Cu, Ag, Nb, Pd, Au, Mg, Sb), alloys (Fe/Cu, Fe/Zn), and oxides (CdO, Fe_2_O_3_), which have been employed for heterogeneous catalysis, solar energy conversion, or as chemical sensors.^15,22,25,26^ Among the aforementioned application domains, spark ablated semiconducting materials would have great potential for the development of a novel approach to further enhance the photocatalytic and photothermocatalytic performances. Despite that, few studies have been devoted to the evaluation of spark-ablated nanoparticles for photocatalytic applications. For example, spark-ablated nanoparticles have been used for aerosol catalysis, which was developed as a tool to study the behaviour of catalyst particles with regard to their characteristic properties such as size, morphology, and surface conditions.^27^ This approach is, however, mainly suitable for analytic purposes, while practical photocatalytic applications would require catalyst immobilization on a supporting material.^28^ In this regard, spark ablation has been employed to produce functional textiles with antimicrobial activity through aerosol deposition of spark-ablated silver nanoparticles.^29^ To the best of our knowledge, spark-ablated nanoparticles have never been evaluated for application in photocatalysis, *i.e.* UV and/or visible-infrared driven photocatalytic decomposition of airborne organic pollutants. In addition, owing to the continuous character of the spark ablation process, nanoparticle generation can beneficially be coupled to direct nanoparticle deposition onto filter media. Photocatalytic filters can thus be manufactured in a single process where the deposition is based on Brownian motion and van-der-Waals-force adherence.^15^

In the present study, one-step production of two types of materials was demonstrated employing the spark-ablation method simply by selecting different electrode materials. First, solar light active MnO_2_ nanoparticles, and second, the traditional UV active photocatalytic nanoparticles, *i.e.* TiO_2_ and ZnO, were generated and directly deposited onto glass fiber filters. The bespoken functionalized filters were calcined under an air atmosphere in order to improve the crystallinity and oxidation states of the materials. After that, the filter samples were characterized and evaluated for the simulated solar light-driven photo(thermo)catalytic and UV photocatalytic performance. In addition, their catalytic performances and stabilities were compared to the sol–gel prepared MnO_2_ and commercially available TiO_2_ and ZnO nanoparticles. It was proven that the spark-ablation method generates photocatalytic nanoparticles of unique properties resulting in superior catalytic performances and stability in comparison to the nanoparticles produced by sol–gel and commercial products.

## Experimental

### Characterization

XRD (X-ray diffraction) analysis of coated filters was performed using a PANalytical X'Pert PRO diffractometer (PANalytical, Netherlands) equipped with a Johansson monochromator (Cu Kα_1_ radiation, *λ* = 1.5406 Å). Raman spectroscopy of coated filters was performed using a Raman spectrometer LabRAM HR Evolution UV-vis-NIR, Horiba, Japan. The Raman analysis was executed employing a 532 nm laser with an output power of 1500 mW. Electron microscopy (EM) images were recorded on a field-emission scanning electron microscope FEI Nova NanoSEM 230 (FEI, USA) with an accelerating voltage of 10 kV and a working distance of ∼5 mm. Carbon coating was applied before the imaging to avoid surface charging. For transmission electron microscopy (TEM) analysis, the coated filter was dropped into methanol and treated in an ultrasonic bath to detach the powder from the filter fibers. The detached nanoparticles were dispersed in methanol and dropped on a lacey carbon film copper TEM grid. The TEM grid with the sample droplet was dried on a heating plate at 80 °C. TEM images and elemental mappings were recorded using high-resolution scanning transmission electron microscopy (HRTEM) with a camera (Gatan Ultrascan, USA) equipped with STEM, BF, and UDF detectors and energy dispersive X-Ray (EDX; JEOL) on a JEOL JEM2200FS microscope operating at 200 kV. In the TEM images, the diameters of spark-ablated nanoparticles were measured using imaging software (ImageJ) and the statistical analysis was performed for the determination of nanoparticle size distributions. In addition, selected area electron diffraction (SAED) was performed for the analysis of the crystallite structure of spark-ablated nanoparticles. The *d*-spacing values were obtained from the measurement of the SAED ring diameters using ImageJ software. The *d*-spacing values were then compared with the standard values of the corresponding Miller indices (*hlk*) and with the values obtained from an online indexing tool (https://www.odpin.com/). Optical properties were analyzed using a UV-vis-NIR spectrophotometer (UV-3600, Shimadzu). The transmittance and reflectance of the coated glass fiber filters were measured using a UV-vis-NIR with a high-performance double monochromator over the wavelength range of 300 nm to 1500 nm, which was enabled by three detectors (photomultiplier tube (PMT), In-GaAs, and cooled PbS). BaSO_4_ was used as a baseline and spectralon was used as a reference for reflectance. Spectral absorbance was determined as absorbance = 1 − (reflectance + transmittance). The Tauc plot and first derivative analysis of the absorption spectra were employed to determine the absorption edges and optical band gaps.^30–32^ X-ray photoelectron spectroscopy (XPS) was performed using a Physical Electronics Quantum 2000 instrument with a monochromatic Al Kα radiation source with an energy of 1486.6 eV. Charge neutralization was accomplished with a dual beam charge neutralization system, using low energy electron and argon ion beams (1 V bias, 20 μA current). XPS survey spectra (BE ranging from 0 to 1100 eV) were recorded with a step size of 0.5 eV at a constant pass energy of 117 eV (power 47.9 W and beam diameter ≈200 μm). High-resolution spectra of the main constituents were recorded with a step size 0.13 eV at a constant pass energy of 58.7 eV. All spectra were calibrated with the C 1s peak of adventitious carbon at 284.8 eV and fitted with mixed Gaussian–Lorentzian components after Shirley background subtraction using CasaXPS software. The peaks in the corresponding core levels of Mn and Ti components were fitted following the empirical models provided by Ilton *et al.*^33^ and Oku *et al.*,^34^ respectively. The area of the corresponding peaks was used for the estimation of the average surface oxidation state of materials.

### Spark-ablated nanoparticle production

**Fig. 1:**
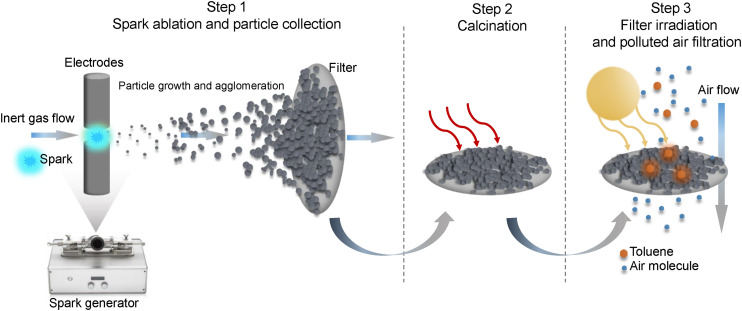
Illustration of the nanoparticle spark generation process followed by particle collection onto filter media (step 1). Step 2 represents the calcination of coated filters in an air atmosphere at 350 °C for MnO_*x*_ Sp, and at 450 °C for TiO_2_ Sp and ZnO Sp. Step 3 illustrates the application of coated filters for the photocatalytic degradation of toluene.

The preparation of samples and the photocatalytic evaluation consisted of three steps as illustrated in Fig. 1, *i.e.* the generation of nanoparticles using a spark generator followed by direct nanoparticle deposition on a filter, calcination in an air environment to improve oxidation, and irradiation of samples in a photocatalytic reactor. In the first step, manganese oxide, titanium dioxide, and zinc oxide (denoted as MnO_*x*_ Sp, TiO_2_ Sp and ZnO Sp, respectively) were produced using the spark ablation (Sp) method. In this process, electrical discharges are initiated between two electrodes made from manganese (95% purity), and titanium and zinc both of 99.99% purity (dimensions of the electrodes: 6 mm diameter and 27 mm length, ChemPur, Germany). The electrodes are mounted onto the holders co-axially, facing each other. Repetitive sparks are induced in the gap between them, each of which forms a vapor cloud of the electrode material, which rapidly condenses into nanoparticles that have the same composition as the electrodes.^35^ The produced nanoparticles are carried away from the gap by a stream of carrier gas (argon, 99.999% Carbagas, Switzerland). During the manipulation and cleaning of the spark generator, essential protective measures were implemented including the use of a lab coat, gloves, safety goggles, and an FFP3 particulate respirator to ensure adequate protection against potential contact with nanoparticles.

The first step of nanoparticle production was directly coupled with the nanoparticle deposition onto a glass fiber filter (LydAir AX1720HD, Lydall Performance Materials, USA). The spark-ablated MnO_*x*_ and TiO_2_ nanoparticles were generated using a voltage of 1.3 kV and current of 6 mA and spark-ablated ZnO was generated using 1.2 kV. Different flow rates of the carrier gas were used in order to control the nanoparticle sizes (10 L min^−1^, 5 L min^−1^, and 1 L min^−1^), with higher flow rates producing smaller particles. The corresponding samples prepared with the different flow rates are denoted as Sp10, Sp5, and Sp1, respectively. A VSParticle model G1 was used for the production of MnO_*x*_ and TiO_2_.^21^ This generator, Mn and Ti electrodes, and a filtration unit were provided by VSPARTICLE B.V. (The Netherlands). Spark-ablation of Zn electrodes was performed using a lab-scale spark-generator (TU Delft). The filters were weighted before and after the spark-ablation process in order to determine the mass of loaded nanoparticles. In the second step, the coated filters with spark-ablated nanoparticles were calcined at 350 °C, 450 °C, and 450 °C for 14 hours in air at normal pressure (Nabertherm N3/R Muffle Furnace, Germany). The main criterion to determine the calcination temperature was the thermal stability of a substrate filter. The employed glass fiber filters can usually withstand operating temperatures up to 500 °C. Based on the literature review, the calcination temperature 450 °C was selected for TiO_2_ materials. In the case of MnO_*x*_, preliminary calcination experiments to prepare the catalysts for toluene degradation were conducted using 150, 350, and 450 °C (Fig. S1†). While the MnO_*x*_ sample was burnt at 450 °C, 350 °C resulted in the highest degradation rate, and thus, this calcination condition was selected. The duration of calcination of 14 h was set based on the literature review and workplace accessibility. During calcination, the particles are oxidized to their final oxidation level whereas some oxidation has already taken place due to the oxygen impurities in the argon carrier gas. After calcination, the samples were stored under ambient conditions before characterization and photocatalytic tests.

### Photocatalytic evaluation

The evaluation of photocatalytic activity was performed in a stainless-steel two-compartment reactor (Fig. S2†), which was designed according to a previous study.^36^ The filter holder-type reactor contained a borosilicate glass enabling irradiation of a filter sample. For the photocatalytic test, the functionalized filters were sealed between the top and bottom compartments and tested for the degradation of toluene, which was selected as a model pollutant. To test the degradation, a stream of toluene–air mixture (50 ppm toluene, 20% oxygen, rest nitrogen, Carbagas, Switzerland) was mixed from a gas tank into the reactor so that a steady-state concentration of around 25 ppm toluene was reached after the closure and circulation of gas in the photocatalytic system. The concentration of toluene was monitored with a gas chromatograph GC-2014 (Shimadzu, Japan) equipped with an Rtx-Wax separation column and an FID-2014 (Shimadzu, Japan) detector. MnO_*x*_ Sp samples were irradiated using a halogen lamp simulating the solar spectrum with an intensity of 222 mW cm^−2^ (equivalent to 1.6-fold intensity of the sun, referring to an annual average of solar irradiation). TiO_2_ Sp and ZnO Sp samples were irradiated with six UV black light bulb (BLB) lamps (368 nm) emitting a total intensity of 0.6 mW cm^−2^. The temperature and relative humidity inside the reactor were approximately 22 °C and 20%. A simplified pseudo-first order kinetic model was applied for the degradation of toluene:^37^

1 ln *C*_0_/*C* = *k*_1_·*t*

where *C*_0_ is the initial concentration of toluene, *C* is the concentration of toluene at a specific reaction time *t* in min and *k*_1_ is the rate constant of the pseudo-first order model in min^−1^. The reaction constant was obtained from the slope of the ln(*C*_0_/*C*) *vs. t* plot. When the coefficient of determination *R*^2^ was larger than 0.95, the experimental data were considered to be in good agreement with this model.^38^ For the determination of degradation constant variability, the error bars in the presented graphs indicated standard errors. The standard errors of 8% and 9% were estimated from the duplicate of toluene degradation experiments using two identical spark-ablated samples of MnO_*x*_ Sp5 and TiO_2_ Sp5, respectively. For sol–gel MnO_2_ and commercial TiO_2_, triplicates of toluene degradation tests showed the standard errors of 8% and 14%. For ZnO samples, the standard error of 8.5% was assumed.

In addition, a comparison with the traditionally prepared photocatalytic materials was performed. MnO_*x*_ nanoflakes were produced by the sol–gel method following a previously reported procedure.^6^ Commercially available TiO_2_ and ZnO nanoparticles used for comparison were purchased from Sigma Aldrich (titanium(iv) oxide nanopowder, 21 nm primary particle size (TEM), ≥99.5% trace metals basis) and from VWR International GmbH (zinc oxide, NanoArc ZN-0605). In addition, ZnO nanorods were produced following a procedure from Zhong *et al.*^39^ These nanoparticles were suspended in demineralized water with 1.5 wt% and sonicated for at least 30 minutes. After that, the filter medium was dipped into the suspension for 10 minutes, withdrawn, dried, and stored under ambient conditions. Such prepared filters were tested in the same photocatalytic configuration as the spark-ablated samples.

## Results and discussion

### Characterization of spark-ablated nanoparticles

#### Crystalline phase and particle structure and size

**Fig. 2:**
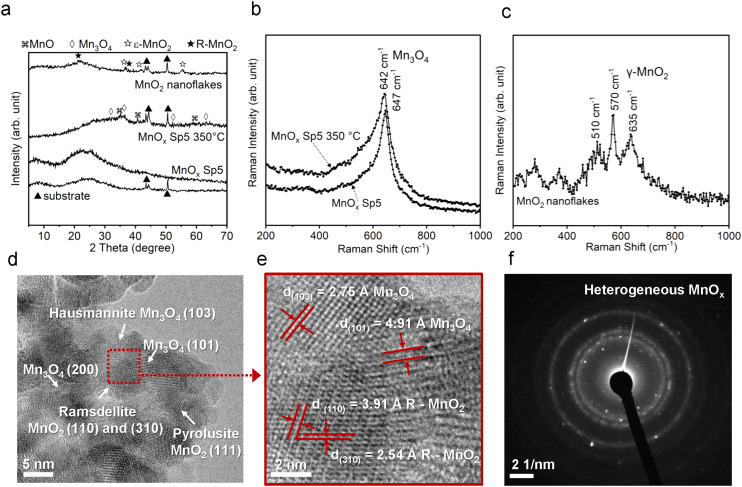
a) XRD diffraction patterns of as-prepared spark-ablated manganese oxide (MnO_*x*_ Sp5), calcined at 350 °C (MnO_*x*_ Sp5 350 °C) and sol–gel prepared MnO_2_ nanoflakes. b) Raman spectra of as-prepared (MnO_*x*_ Sp5) and calcined (MnO_*x*_ Sp5 350 °C) spark-ablated samples. c) Raman spectrum of the sol–gel prepared MnO_2_ nanoflake sample. d) HRTEM images of MnO_*x*_ Sp showing hausmannite, ramsdellite and pyrolusite crystalline structures; e) measured planar spacing with the determination of miller indices (*hkl*). f) Selected area electron diffraction (SAED) image of heterogeneous MnO_*x*_ Sp5.

**Fig. 3:**
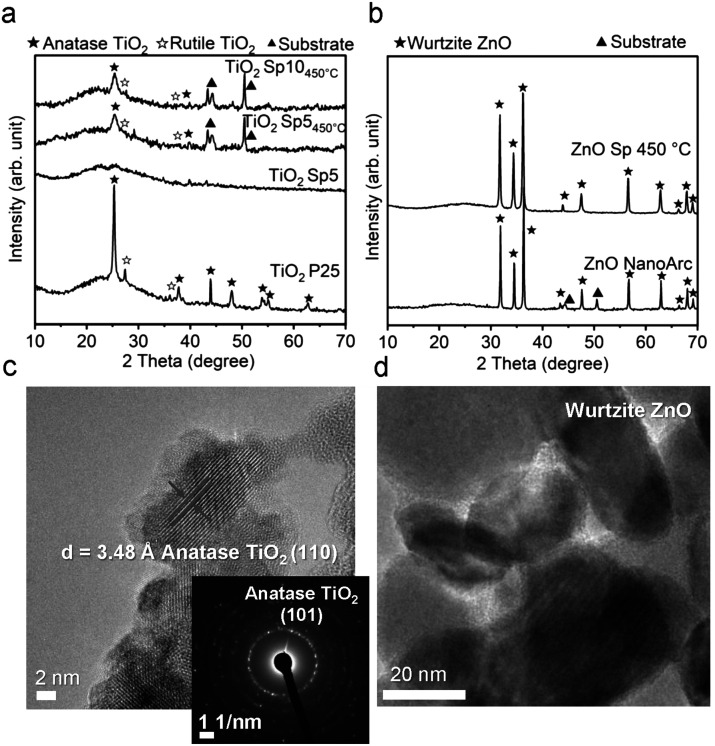
XRD diffraction patterns of a) titanium dioxide (TiO_2_) and b) zinc oxide (ZnO) produced by spark ablation in comparison with calcined and commercial samples. c) HRTEM image of TiO_2_ Sp5 displaying the anatase crystalline structure and its corresponding selected area electron diffraction (SAED) image. d) HRTEM image of ZnO Sp nanoparticles.

The XRD patterns of spark-ablated nanoparticles were used to determine the crystalline phase of the MnO_*x*_, TiO_2_, and ZnO nanoparticles (Fig. 2 and 3). The strong diffraction patterns at 43, 44, and 50 degrees originated from the filter substrate. All samples showed weak XRD diffraction patterns, and thus, crystalline structure identification was challenging. This is attributed to the small nanoparticle size with randomly oriented diffraction planes and the porous structure of spark-ablated nanoparticle deposition, which results in a low signal-to-noise ratio, making proper identification of the crystallite phase difficult.^15,40^ After calcination, a slight increase in the diffraction pattern intensity was observed for Mn and Ti samples (Fig. 2a and 3a) due to improved crystallinity. The XRD patterns of spark-ablated and calcined MnO_*x*_ and TiO_2_ samples were compared with MnO_*x*_ and TiO_2_ standards. For MnO_*x*_, the produced material contained diffraction pattern peaks of several standards (mainly MnO and Mn_3_O_4_) indicating the heterogeneous crystalline structure, but more specific identification was not possible. In the case of the Ti samples, a clear diffraction peak at around 25 degrees appeared after the calcination, which corresponded to the anatase phase. Due to the significantly larger particle size, ZnO Sp nanoparticles showed strong diffraction patterns, which corresponded to the commercial ZnO sample (Fig. 3c). Both ZnO samples were identified as the hexagonal wurtzite crystalline structure, which was the most common structure owing to its stability under ambient conditions.^41^

In order to elucidate the local crystal structure and its changes after the calcination of the spark-ablated MnO_*x*_ sample, Raman spectroscopy was conducted. The Raman analysis was executed employing a 532 nm laser with an output power of 1500 mW. Recognizing the potential susceptibility of manganese oxides to laser-induced degradation during Raman analysis,^42^ we systematically explored four discrete power conditions, specifically 0.1%, 1%, 5%, and 10% of the full laser power (as illustrated in Fig. S3†). The test aimed to ascertain the most optimal parameters for mitigating sample degradation. The results indicated that a laser power setting of 1%, corresponding to 15 mW, in combination with a 100× magnification and an accumulation time of 60 seconds, effectively minimized sample degradation. Consequently, these parameters were selected for subsequent evaluation. Notably, the Raman spectra of the spark-ablated MnO_*x*_ samples, both in their as-prepared and calcined states, exhibited similarities. These Raman spectra displayed relatively weak bands at approximately 260 cm^−1^, 330 cm^−1^, 410 cm^−1^, 464 cm^−1^, and 564 cm^−1^, as well as a pronounced band at 640 cm^−1^. The bands situated at approximately 560 cm^−1^ and 640 cm^−1^ are characteristic of the stretching vibration of Mn–O–Mn and Mn–O, respectively, where the strong vibration at 640 cm^−1^ could be associated with the Mn–O stretching vibration of Mn^2+^ in tetrahedral coordination within the hausmannite Mn_3_O_4_ phase.^42,43^ In contrast, the sol–gel prepared MnO_2_ sample unveiled a distinct Raman spectrum, featuring bands at 220 cm^−1^, 279 cm^−1^, 370 cm^−1^, 510 cm^−1^, 570 cm^−1^, 635 cm^−1^, 680 cm^−1^, and 740 cm^−1^. The pronounced vibration at the band above 500 cm^−1^ is attributed to the internal MnO_6_ octahedral Mn–O stretch and O–Mn–O bend modes, where the complex Raman spectrum showing multiple peaks reflects a lower symmetry of the Mn–O octahedral environment of the ramsdellite matrix with pyrolusite intergrowths.^44^ In addition, a comparative evaluation of Mn–O vibrational frequencies at around 640 cm^−1^ of the as-prepared and calcined samples revealed pronounced broadening and redshifting from 647 cm^−1^ to 642 cm^−1^ subsequent to the calcination (Fig. S4†). This phenomenon suggested lattice expansion stemming from an augmented presence of oxygen vacancies caused by the calcination process.^43^ Remarkably, this broadening was even more pronounced compared to the sol–gel prepared MnO_2_ sample, indicating a more substantial lattice distortion attributable to a higher concentration of oxygen vacancies.

To further investigate the microstructure of spark-ablated nanoparticles, the deposited nanoparticles were collected from the collection filters and examined with high-resolution transmission electron microscopy (HRTEM) and selected area electron diffraction (SAED) analysis (Fig. 2d–f). HRTEM image analysis of interplanar distances confirmed the heterogeneous nature of MnO_*x*_ SP, where the lattice structure was determined using the standard relation connecting the interplanar distance (*d*) of samples with standard values of interplanar distances and Miller indices (*hkl*). The results revealed the presence of three MnO_*x*_ polymorphs: hausmannite Mn_3_O_4_, ramsdellite and pyrolusite MnO_2_ (Fig. 2d and e). The intergrowth domains of ramsdellite and pyrolusite varieties are generally assigned to γ-MnO_2_.^45^ The corresponding SAED data showed bright diffraction rings, which demonstrated the formation of polycrystalline MnO_*x*_ consisting of the three polymorphs (Table S1†). This shows that spark-ablation synthesis in argon followed by calcination in air at 350 °C for 14 h led to the formation of mixed-valence Mn_3_O_4_ and γ-MnO_2_. For TiO_2_ SP, the HRTEM and SAED analyses were used to confirm the anatase crystalline structure, as shown in Fig. 3c and Tables S2.†

The findings from XRD, Raman spectroscopy, HRTEM and SAED demonstrated that the spark ablation method produced hausmannite Mn_3_O_4_ as the predominant crystalline structure, which is a stable manganese phase at high temperature.^46^ The formation of the hausmannite phase is favored due to the rapid heating during the spark ablation of the electrode material where the temperature of local “hot spots” can reach 10 000 K.^26^ Similarly, the formation of MnO and Mn_3_O_4_ has been reported to be thermodynamically favored in oxygen-lean high-temperature processes such as the flame and laser ablation syntheses.^47,48^ This would suggest that the Mn electrode is vaporized to form mainly the Mn_3_O_4_ phase in the stream of the oxygen-lean carrier gas. The subsequent calcination of the Sp sample led to the increased crystallinity as shown by the increase of Raman intensity (Fig. 2b). In the course of spark ablation of TiO_2_, the XRD analysis revealed that the spark ablation method produced mainly the amorphous Ti phase, which was transformed to anatase during the calcination. The presence of anatase was further confirmed by HRTEM and SAED analysis. This preference for anatase formation can be ascribed to the specific particle size conditions and the calcination temperature below 600 °C, where anatase represents the thermodynamically stable crystalline phase.^49^

**Fig. 4:**
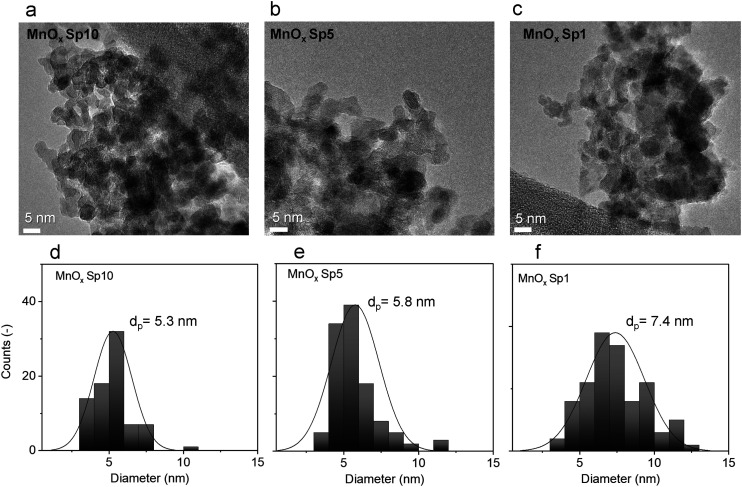
TEM images of a) manganese oxide (MnO_*x*_) Sp10, b) MnO_*x*_ Sp5, and c) MnO_*x*_ Sp1 spark-ablated nanoparticles using 10 l min^−1^, 5 l min^−1^, and 1 l min^−1^ flow rates, respectively, for nanoparticle generation and deposition; d)–f) the corresponding primary particle size distributions.

**Fig. 5:**
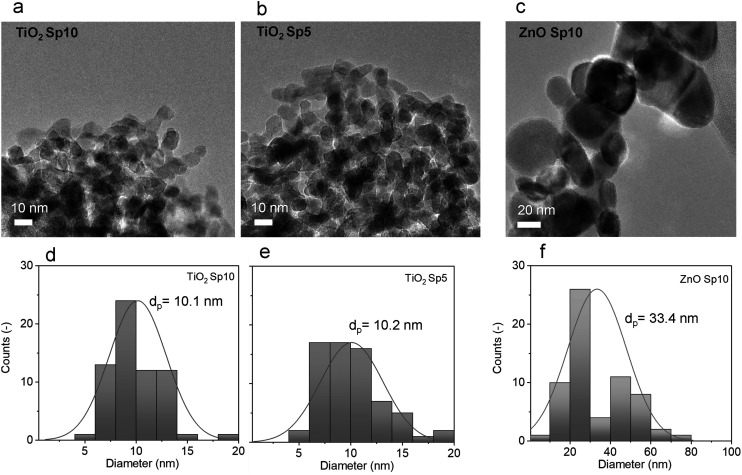
TEM images of a) titanium dioxide (TiO_2_) Sp10, b) TiO_2_ Sp5, and c) zinc oxide (ZnO) Sp10 spark-ablated nanoparticles using 10 l min^−1^ (Sp10) and 5 l min^−1^ (Sp5) flow rates, for nanoparticle generation and deposition; d)–f) the corresponding particle size distributions.

Furthermore, TEM images were used for nanoparticle size analysis. The TEM images of the Sp samples showed sphere-like primary particles with relatively narrow normal, approximately Gaussian, size distributions. The particle size increased with decreasing flow rate of the carrier gas. The count median diameters (CMD) of MnO_*x*_ Sp nanoparticles were 5.3 nm, 5.8 nm, and 7.4 nm for 10 L min^−1^, 5 L min^−1^, and 1 L min^−1^ flow rates, respectively (Fig. 4). For TiO_2_, the sizes were 10.1 nm and 10.2 nm for 10 L min^−1^ and 5 L min^−1^ flow rates, respectively (Fig. 5). The size of ZnO Sp nanoparticles was determined as 33.4 nm with a wide size distribution for the flow rate of 10 L min^−1^.

The effect of different flow rates on the particle size is attributed to the increased cooling rate and transport velocity of primary particles with a higher flow rate. This reduces sintering and coalescence between primary particles and results in a smaller particle size and narrower size distributions.^50^ In particular, metal particles show complete coalescence (*i.e.* they retain a sphere-like shape) during the first stage of growth.^20^ At a given temperature, there is a maximum size for complete coalescence to take place. The so-called primary particle size (the size of the smallest “round” structures observed in an agglomerated structure) depends on the coalescence behavior of the material (bulk and surface diffusion of the atoms) under their temperature history. For the present case, where the metal is (partially) oxidized during particle growth by O_2_ impurity in the carrier gas and the composition changes continuously, the process is complex and rather unpredictable. The primary particles form agglomerates in the gas phase and continued to agglomerate on the substrate. As oxidation is likely not complete when agglomeration begins, we have to assume that it continues while the primary particles are in contact in the aerosol phase and after deposition on the filter fibre. This chemical conversion leads to the displacement of atoms in the nanostructured solid, which, together with the thermodynamic driving force towards surface minimization, may lead to further sintering and coalescence. This phenomenon, which can be referred to as “oxidation-induced coalescence”, has been observed during the oxidation of iron nanoparticles.^51^ To our knowledge, this phenomenon has barely been studied systematically.

**Fig. 6:**
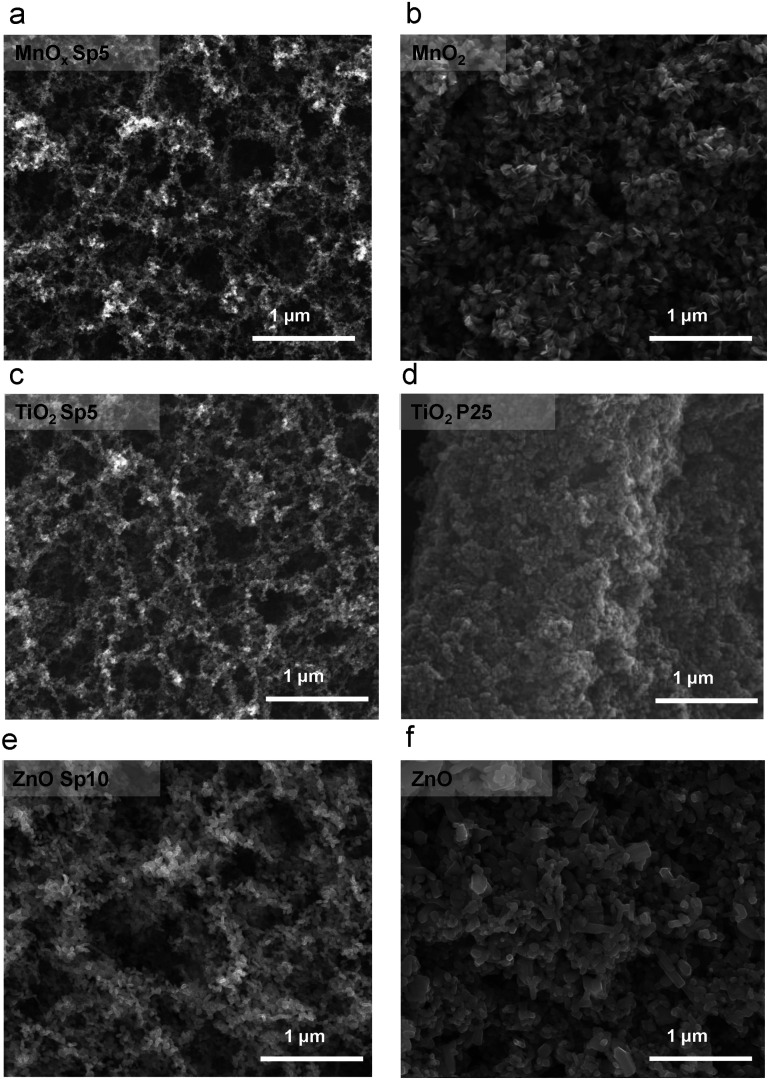
SEM images of spark-ablated nanoparticles and nanoparticles prepared by the sol–gel method for a) and b) manganese oxide (MnO_*x*_), c) and d) titanium dioxide (TiO_2_), and e) and f) zinc oxide (ZnO), respectively.

The size range of collected nanoparticles indicated that diffusion was the dominant mechanism of deposition, especially for MnO_*x*_ and TiO_2_ Sp with sub-20 nm sizes. Structures with high surface-to-volume ratio and high porosity are formed during the particle transport dominated by Brownian motion,^15^ which is observed by scanning electron microscopy (SEM) imaging in our study (Fig. 6). The SEM images provided an overview of the nanoparticles deposited on the glass fibre substrate. Agglomeration in the aerosol phase forms fractal-like agglomerates, which combine to form porous and web-like layers on the filter fibres (Fig. 6). The Sp nanoparticles were smaller and the deposition structure was more porous compared to the sol–gel MnO_2_ and commercially available TiO_2_ and ZnO nanoparticles, which were immobilized by a dip-coating method, where the particles were larger and densely packed on the fibres.

**Fig. 7:**
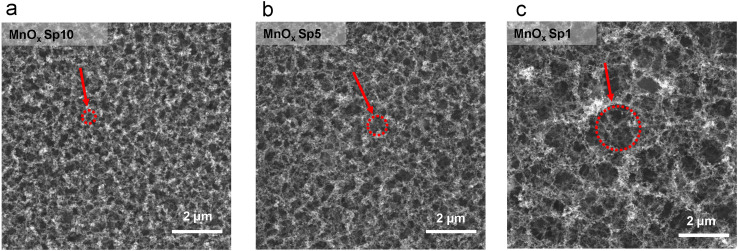
SEM images of manganese oxide spark-ablated (MnO_*x*_ Sp) nanoparticles with carrier gas flow rates of a) 10 l min^−1^, b) 5 l min^−1^, and c) 1 l min^−1^. The red circles highlight different pore sizes originating from different flow rates.

The deposited structures were affected by the different flow rates during Sp nanoparticle generation and deposition. The structures were examined by SEM imaging (Fig. 7), which revealed that the smaller the flow rate, the larger the holes between agglomerated structures (Fig. 7). This can be explained by the fact that under slow flow, there is more time for agglomerate growth in the gas phase, and the resulting larger agglomerates leave more void space between each other after deposition.^50,52^

#### Optical properties

At the nanoscale, occupied electronic states above the valence band of the corresponding material expand the physical and chemical activities of the systems. Therefore, it is essential to understand the optical properties and band-gap characteristics of produced materials as they play an important role in photocatalytic processes. The absorption spectra of spark-ablated and calcined nanoparticles were collected using UV-vis-NIR spectroscopy.

**Fig. 8:**
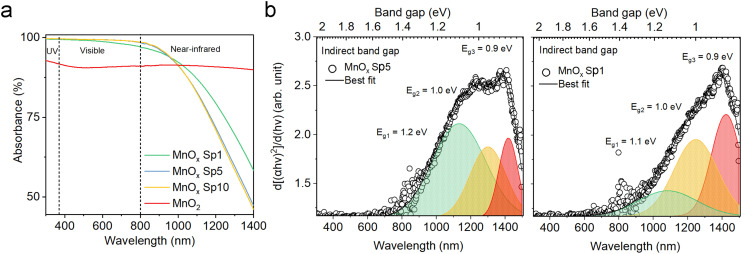
Absorption properties of spark-ablated particles for a) manganese oxide (MnO_*x*_) prepared under different conditions and b) the estimation of indirect optical band gap using the 1st derivative of the Tauc plot for MnO_*x*_ Sp5 and Sp1 samples with the fits of three levels of transitional energy.

Dark-brownish MnO_*x*_ Sp samples (Fig. S5a†) demonstrated superior absorption in the UV-vis spectrum, while the absorption decreased in the NIR spectrum (Fig. 8a). The absorption spectra were similar for MnO_*x*_ Sp10 and Sp5 samples, while Sp1, *i.e.* the larger particles, showed higher absorption in the NIR spectrum. Using a Tauc plot of the absorption spectra, a band gap of 1.2 eV was estimated for Sp10 and Sp5 samples while a slightly lower band gap of 1.0 eV was observed for the Sp1 sample (Fig. S6a†). Band gap values between 1 eV and 2 eV have been reported previously for MnO_2_.^53,54^ In addition, the 1st derivative of the Tauc plot (Fig. 8b) showed that the optical transition from the valence-band electronic structure contained three levels of transitional energy, which accounted for the hausmannite, ramsdellite, and pyrolusite phases of the MnO_*x*_ Sp sample.^32^ The present analysis also revealed that the composition changed for the lowest flow rate, *i.e.* the largest particle size. This could be attributed to the different kinetics of oxidation and coalescence under the low-flow-rate spark ablation process. The sol–gel MnO_2_ sample showed a constant absorption with no transitional energy observed in the UV-vis spectrum (Fig. 8a and S6b†).

**Fig. 9:**
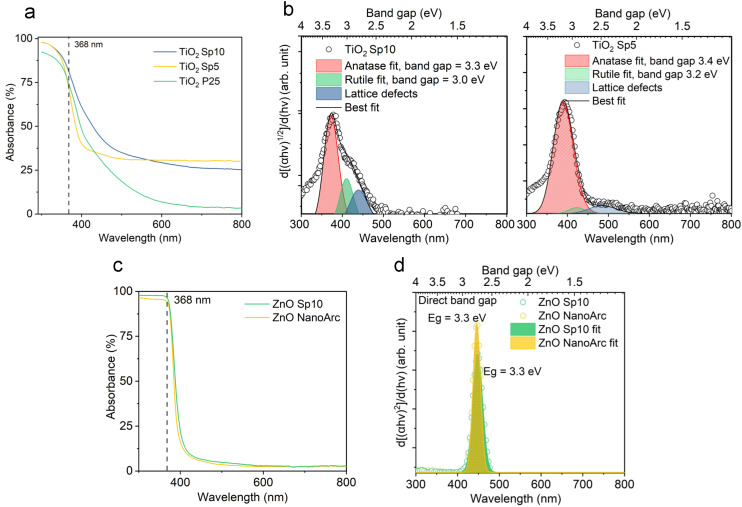
a) Absorption properties and indirect optical band energy obtained using b) the 1st derivative of the Tauc plot with the fits of optical band transition energy levels for titanium dioxide (TiO_2_) anatase and rutile phase Sp10 and Sp5 samples. c) Absorption and d) the 1st derivative of the Tauc plot for zinc oxide (ZnO) spark-ablated and commercial nanoparticles.

The colors of the just-produced TiO_2_ Sp and ZnO Sp samples were grey and brown, respectively. During the calcination, the color changed from grey to light grey and from brown to white (Fig. S5b–d†). The color of pure TiO_2_ and ZnO materials is usually white with absorption in the UV spectrum and their color change is determined by various factors, such as the size of particles, the level of crystallinity, and the existence of defects and impurities. In addition, a microscopic inspection of the spark-ablated nanoparticles before and after calcination was performed using SEM (Fig. S7 and S8†). Our observations revealed no discernible alterations in the size or structure of the deposited nanoparticles. Consequently, we assumed that the observed color change was associated with variations in the oxidation state and the quantity of surface defects. The distinct color of just-produced Sp samples accounted for a low level of crystallization and oxidation, which was further improved after the calcination that induced color blending.^55^ The calcined TiO_2_ Sp samples showed a red shift and increased absorption in the UV-vis spectrum (Fig. 9a). The shift and increased absorption are enabled by the delocalization of molecular orbitals at the conduction band edge, which generates shallow and/or deep traps within the band gap.^30^ In the present study, the band gap characteristics were studied using a commonly used Tauc plot analysis and a more sensitive 1st derivative approach. Using the Tauc plot analysis, the band gaps of the Sp and commercial materials were estimated to be 3.2 eV for TiO_2_ Sp10 and TiO_2_ Sp5 compared to 3.1 eV for TiO_2_ P25 (Fig. S6c†), which corresponded well to the reported values.^56^ While Sp5 showed a relatively sharp absorption edge, the Sp10 sample showed a gradual decrease of absorption at energies below the determined band gap so-called Urbach tail (Fig. S6c†).^5^ This additional broad absorption band is a phenomenon caused by the presence of defects, such as Ti^3+^ interstitials and oxygen vacancies.^57^ For the Sp10 sample, the presence of defects was further observed in the 1st derivative representation as an additional transition peak at an energy around 2.7 eV (Fig. 9b). The differences in the defect amount and the degree of oxidation were also apparent from the different hues of grey before and after calcination of the Sp10 and Sp5 samples (Fig. S5b and c†). This can be explained by the higher flow rate of the carrier gas during the spark ablation of this sample. In general, the higher flow rate increases dilution and reduces the likelihood of oxidation or other chemical reactions, which can influence the level of oxidation and the amount of bulk and surface defects such as Ti interstitials and oxygen vacancies in the TiO_2_ structure.^26,55^ The additional peak at around 2.7 eV, as well as the aforementioned Urbach tail, was also observed to some extent in the commercial TiO_2_ sample (Fig. S6c and d†). As the color of the commercial TiO_2_ was white, this observation can be attributed mainly to the presence of impurities and adsorbed hydroxyl groups. Further inspection using the 1st derivative of the Tauc plot revealed two levels of transition energy at 3.3 eV and 3.1 eV representing typical values for the anatase and rutile phases of TiO_2_.^56^ The fitting analysis revealed the different anatase/rutile compositions of the TiO_2_ material (Fig. 9b). While TiO_2_ Sp10 contained about 15% of rutile, the Sp5 sample consisted of mainly anatase crystalline phases. This observation is in accordance with the XRD pattern intensity comparison (Table S3†). Even though the anatase phase is usually metastable, it becomes more stable than the rutile phase at the nanoscale due to its smaller surface energy.^58^ The transformation of anatase to rutile is enabled during the calcination above 600 °C, which exceeds the 450 °C used in our study by far.

The samples of ZnO SP, on the other hand, showed optical properties very similar to those of the commercial ZnO. Just a slight increase in the UV absorption along with a red shift was observed from the absorption spectra (Fig. 9c) and the optical band gap was reduced slightly for the ZnO Sp sample (Fig. 9d and S6e†). Both samples showed one transition energy accounting for the wurtzite phase. Similar to TiO_2_, the red shift and a slight reduction of the optical band gap of ZnO materials are attributed to subband transitions closely associated with the concentration of oxygen vacancies at the surface.^59^

#### Surface properties

**Fig. 10:**
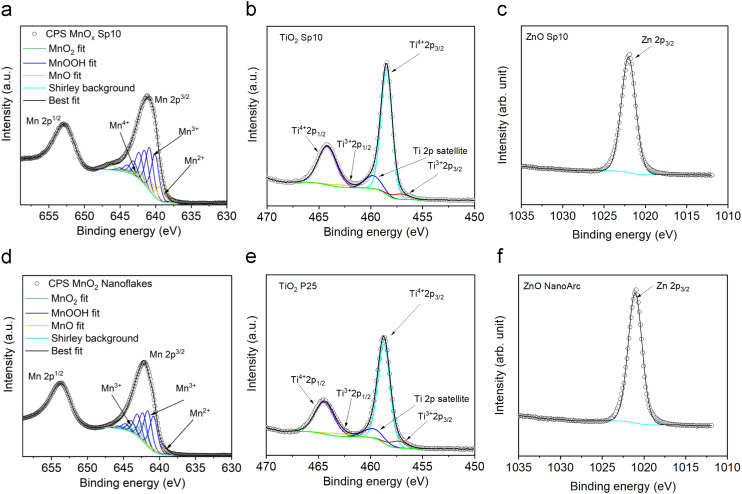
High-resolution a) Mn 2p, b) Ti 2p, and c) Zn 2p XPS spectra of spark-ablated manganese oxide (MnO_*x*_), titanium dioxide (TiO_2_), and zinc oxide (ZnO) nanoparticles, respectively, and the comparison with the XPS spectra of d) sol–gel prepared MnO_2_ and commercial e) TiO_2_ P25 and f) ZnO NanoArc. Peak deconvolution indicates the oxidation states of the material.

XPS is a surface-sensitive technique that provides information about the chemical state of materials. Here, the chemical states of Mn, Ti, and Zn with respect to O were analyzed in order to understand the oxidation state of the Sp materials.^60^ The resulting XPS core level of Mn 2p_3/2_ (binding energy 642 eV) was fitted with MnO, MnOOH, and MnO_2_ standards in order to determine the proportion of Mn^2+^, Mn^3+^, and Mn^4+^ (Table S4†). It has been reported that the MnOOH standard provides the best fits for Mn^3+^ proportion determination of MnOOH, Mn_2_O_3_ and Mn_3_O_4_.^33^ The average oxidation states (AOSs) of produced Sp nanoparticles were determined as 2.9 for Sp10 and Sp5, and 2.8 for Sp1 accounting for about 20% Mn^2+^, 70% Mn^3+^, and 10% Mn^4+^ (Fig. 10). The determined AOS and composition are different from the theoretical AOS value of 2.66 for Mn_3_O_4_ corresponding to 66.7% Mn^3+^ and 33.3% Mn^2+^ (ref. 33 and 61) and the AOS value of 4 for MnO_2_. This confirms the multivalence of MnO_*x*_, which consists of the major phase of Mn_3_O_4_ with a minor contribution of MnO_2_. In addition, the multiplet splitting of Mn 2p and Mn 3s XPS levels is usually used for the characterization of the MnO_*x*_ valence. The multiplet splitting values of the Sp samples were determined as 11.8 eV for Mn 2p and 5.6 eV for Mn 3s (Fig. S9†), following the reported values for Mn_3_O_4_.^33,61^ These results confirm the observation of the crystalline structures from SAED analysis. The AOS of sol–gel-prepared MnO_2_ was 3.2 with a Mn 3s multiplet splitting of 4.9 eV (Table S4†) indicating that the sol–gel sample also contained a significant amount of reduced manganese (Mn^3+^) on the surface. The AOS of sol–gel MnO_2_ is close to the reported values of the surface AOS for MnO_2_ (AOSs of 3.3–3.9).^62^ It is evident that different preparation methods introduce different oxidation states and vacancies.

In the same manner, the TiO_2_ Sp sample was fitted with Ti^3+^ and Ti^4+^ standards.^34^ The obtained value of the AOS was 3.8, similar to the commercial TiO_2_ sample. These results show the tendency of the Ti material to be stable in the highest oxidation state, though a small amount of reduced Ti^3+^ was present on the surface. Spark-ablated and commercial ZnO samples showed similar oxidation states. The binding energies of Sp ZnO and TiO_2_ were slightly shifted in comparison to the commercial samples, which indicated differences in the surface properties. The Ti 2p_3/2_ binding energy of the TiO_2_ Sp sample was shifted to lower energy from 458.7 eV to 458.5 for Sp5 and 458.3 for Sp10 eV, which might be assigned to Ti^3+^ in the TiO_2_ lattice.^63^ The Zn 2p_3/2_ core level of the ZnO Sp sample showed a shift to higher binding energy from 1021.8 eV to 1022.1 eV. This observation is closely related to the concentration of lattice defects and oxygen vacancies.^64^

**Fig. 11:**
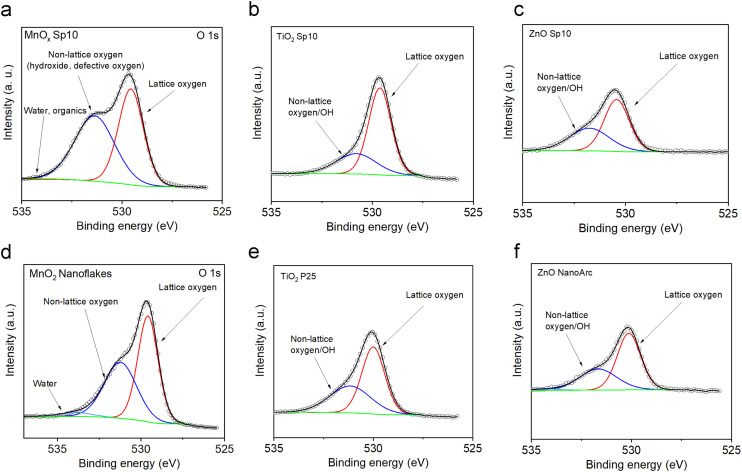
High-resolution O 1s XPS spectra of spark-ablated a) manganese oxide (MnO_*x*_), b) titanium dioxide (TiO_2_), and c) zinc oxide (ZnO) nanoparticles, respectively, and the comparison with O 1s XPS spectra of d) sol–gel prepared MnO_2_ and commercial e) TiO_2_ P25 and f) ZnO NanoArc. Peak deconvolution indicates the presence of lattice oxygen, non-lattice oxygen and/or adsorbed OH groups, and adsorbed water.

Using fits of the O 1s core level, the ratios between lattice and non-lattice oxygen were determined to investigate the surface defects and oxygen vacancies (Fig. 11).^60,64^ The data revealed that MnO_*x*_ Sp nanoparticles contained up to 52% of non-lattice oxygen/surface defects and the highest fraction of non-lattice oxygen (*i.e.* hydroxyl group, oxygen vacancies, and/or surface defects) was found in the spark-ablated nanoparticles with the smallest size (Fig. 11a and Table S4†). The larger amount of surface defects is ascribed to (i) the small size, (ii) the presence of impurities in the carrier gas, and (iii) the low calcination temperature.^65^ This observation confirmed the results from Raman spectroscopy (Fig. S4†) where a broadening of the Mn–O vibrational mode at around 640 cm^−1^ indicated a high level of lattice distortion. TiO_2_ Sp contained less non-lattice oxygen along with higher AOS values accounting for a less defective surface (24% and 30% of non-lattice oxygen for Sp10 and Sp5 samples, Fig. 11b and Table S5†). This observation is different from the UV-vis spectrometry observation, where the Sp10 sample showed the highest amount of defects through the Urbach tail in the Tauc plot representation (Fig. S6b†). As XPS is a surface-sensitive technique while UV-vis spectrometry can probe bulk properties of materials, this indicates that the TiO_2_ Sp samples contained mainly bulk defects, which persisted even after the calcination.^66^ The ZnO Sp sample contained 41% of non-lattice oxygen (Fig. 11c, Table S5†) and the non-lattice oxygen fractions of the sol–gel MnO_2_ sample, commercial TiO_2_ P25, and ZnO NanoArc samples were determined as 44%, 41%, and 37%, respectively (Fig. 11d–f and Tables S4 and S5†). It should be noted that the non-Sp samples exhibited different origins of non-lattice oxygen mainly assigned to the surface impurities and adsorbed hydroxyl groups as the preparation of the samples involved an aqueous solution with chemical precursors.

### VOC degradation performance

#### Photocatalytic activity

**Fig. 12:**
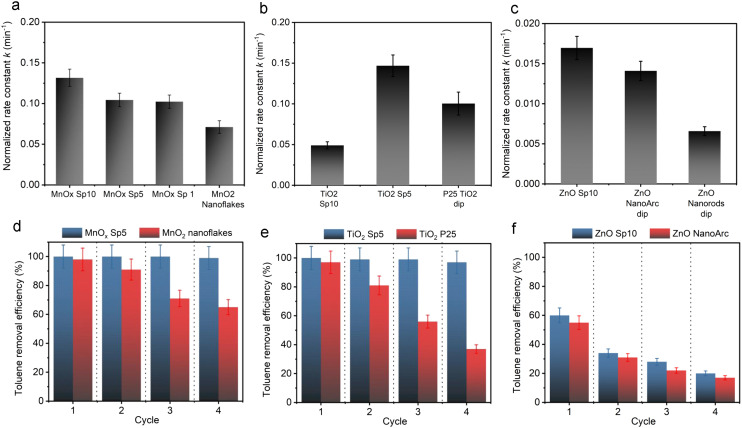
Normalized 1st order reaction rate constant *k* for a) spark-ablated manganese oxide (MnO_*x*_ Sp) nanoparticles and MnO_2_ nanoflakes, b) spark-ablated and commercial titanium dioxide (TiO_2_ Sp and TiO_2_ P25), and c) spark-ablated and commercial zinc oxide (ZnO Sp10 and ZnO NanoArc) nanoparticles. Photocatalyst stability over four consecutive toluene degradation cycles represented as toluene removal efficiency after 60 min of irradiation for d) MnO_*x*_ Sp and MnO_2_ nanoflakes, e) TiO_2_ Sp5 and TiO_2_ P25, and f) ZnO Sp10 and ZnO NanoArc nanoparticles.

The photocatalytic performances of the Sp nanoparticles immobilized on glass fiber filters were evaluated in a photocatalytic reactor (Fig. S2†) using simulated solar light for MnO_*x*_ and UV light for the TiO_2_ and ZnO samples. The decomposition of toluene was considered for the evaluation of photocatalytic activities. The toluene concentration was continuously monitored through GC-FID. The toluene signal exhibited a gradual decline over time without the emergence of additional peaks. The absence of such peaks suggested the absence of detectable intermediates in the gas phase, given that the concentrations remained below the GC-FID detection limit of 100 ppb. This study emphasizes showcasing the photocatalytic proficiency of spark-ablated nanoparticles and subsequently comparing it with catalysts generated through alternative methods. A comprehensive examination of intermediates would necessitate further investigation. Through a comparative analysis of toluene removal efficiencies and the corresponding normalized 1st order kinetic constants, it is evident that all Sp nanoparticles provided enhanced photocatalytic activity in comparison to the sol–gel and benchmarked commercial photocatalysts (Fig. S11†). As photocatalysis and photothermocatalysis are complex processes, the activity is affected by multiple factors, such as the crystalline and electronic structure on the surface and subsurface of the material, and the size of primary particles and their agglomeration. These properties influence light absorption and electron/pair excitation and transfer. Our spark-ablated nanomaterials demonstrated unique properties leading to the improved photocatalytic activity and stability as discussed in the following paragraphs (Fig. 12).

In our study, the smallest nanoparticles were produced by the spark ablation of Mn electrodes. MnO_*x*_ Sp nanoparticles showed superior photocatalytic performance compared to the sol–gel prepared samples, specifically, all Sp samples led to the complete removal of toluene (around 25 ppm) in less than 50 min using a halogen lamp with 222 mW cm^−2^ (Fig. 12a and S11†). The mass-normalized 1st order degradation constants were determined as 0.132 min^−1^, 0.104 min^−1^, 0.103 min^−1^, and 0.071 min^−1^ for Sp10, Sp5, Sp1 MnO_*x*_, and sol–gel MnO_2_ sample, respectively (Table S6†). We ascribed these improvements of the spark-produced samples with respect to the benchmarks to the crystalline structure and the surface properties, *e.g.*, the content of Mn^2+^/Mn^3+^/Mn^4+^ and the oxygen vacancies, which were different for the Sp and sol–gel samples. Specifically, as revealed by XRD and Raman spectroscopy, the spark ablation of Mn electrodes followed by calcination resulted in the formation of Mn_3_O_4_ as the predominant manganese crystalline phase. The presence of additional phases was observed using HRTEM, UV-VIS spectrometry, and XPS analysis. The XPS analysis showed the presence of a greater content of Mn^3+^ with a minor content of Mn^2+^ and Mn^4+^ on the surface of the Sp samples indicating the coexistence of Mn_3_O_4_ with a small portion of MnO_2_. In addition, both Raman spectroscopy and XPS revealed a high level of lattice distortion, *i.e.*, oxygen vacancies, which contributed to the reactivity of Sp samples. Based on the literature review, it has been shown that manganese oxides with mixed valence and the presence of active oxygen promote the photodegradation of organic molecules.^67^ In particular, it has been reported that the oxidation activity of mixed-valence Mn_3_O_4_ is based on a thermo-assisted photocatalytic process.^11^ In this process, during solar irradiation, the d–d orbital electrons of the Mn atom within the [MnO_6_] octahedron absorb photon energy, leading to a transition from the ground state to an excited state, generating electron–hole pairs. This weakens the Mn–O bond and enhances the activation of lattice oxygen activity. As the light exposure continues, the thermal energy generated through the photothermal effect augments photocatalytic performance by increasing charge transfer. Furthermore, this thermal energy contributes to enhancing lattice oxygen activity, generating additional surface-active oxygen, while the remaining oxygen vacancies are replenished by the surrounding O_2_ molecules.^11,68^ The coexistence of various manganese oxides, such as Mn_2_O_3_, Mn_3_O_4_, and MnO_2_, has been reported to be beneficial for improved visible-light-driven organic degradation due to their stronger visible light absorption, more active sites, and enhanced charge separation performance.^10,69^ In such heterojunction nanostructures, the efficient spatial separation and prolonged lifetime of photo-induced carriers (*e.g.*, electrons and holes) can be achieved because the conduction (CB) and valence bands (VB) of Mn_2_O_3_ and Mn_3_O_4_ are more negative than the CB and VB of MnO_2_. The reported valence band energies are 0.34, 1.85, and 2.19 eV, respectively, with corresponding conduction band energies of −1.09, −0.35, and 0.43 eV. This thermodynamically allows the migration of excited electrons from the CB of Mn_2_O_3_ to the CB of Mn_3_O_4_ and to the CB of MnO_2_, effectively extending the lifespan of photo-induced carriers. Superoxide radicals are generated through reactions between oxygen and electrons on the conduction bands of Mn_2_O_3_ and Mn_3_O_4_, which can further react with hydrogen ions to form hydroxyl radicals. Conversely, the photogenerated holes remain in the corresponding VB, with MnO_2_ having the highest conduction band energy of 2.19 eV, where adsorbed hydroxyl ions can be oxidized into hydroxide radicals.^69^ A similar study^70^ focused on the degradation of toluene using an α-MnO_2_/Mn_3_O_4_ hybrid system, showing that this nanostructure promoted the generation of superoxide radicals through interfacial charge transfer and provided more oxygen vacancies for dissociative adsorption of oxygen.^70^ These findings are in line with our observations, where the conduction energies of MnO_*x*_ samples were estimated to be 1.6 eV and 2.0 eV for Mn_3_O_4_ and MnO_2_,^10^ respectively, with corresponding conduction bands of 0.4 and 0.8 eV (Fig. S10†). In addition, it was reported that the small-size particles experience low resistance of interfacial charge transfer, which enhances the photocatalytic activity of heterojunction structures.^10^ The presented results for spark-ablated samples showed that a significant improvement of the degradation rate was observed for the smallest size, *i.e.* MnO_*x*_ Sp10. In addition, this sample provided the largest amount of surface defects as indicated by XPS analysis of the O 1s core level (Table S4†) and Raman spectroscopy (Fig. S4†), which could act as additional active sites, and the highest absorption in the visible light spectrum (Fig. 8). The increased absorption in the visible spectrum and the determined band gap indicated that the degradation on spark-ablated manganese oxide samples was driven by the thermal-assisted photocatalytic process rather than light-drive thermocatalysis. The present study primarily focused on the spark ablation production of various materials to assess the suitability of this procedure for photocatalyst synthesis. It should be noted that the comprehensive characterization and evaluation of the oxidation mechanism using heterogeneous spark-ablated manganese oxide were not within the scope of this investigation. Further exploration of this aspect will be necessary in future studies.

Employing TiO_2_ samples under UV irradiation with an intensity of 0.6 mW cm^−2^, a significant improvement in the toluene degradation rate was observed for TiO_2_ Sp5 (Fig. 12b and S11†). This sample showed almost three times and 1.5 times faster toluene removal in comparison to TiO_2_ Sp10 and commercial TiO_2_, with a mass normalized rate constant of 0.147 min^−1^, 0.049 min^−1^, and 0.100 min^−1^, respectively (Table S6†). The particle sizes in the Sp10 and Sp5 samples were similar, but Sp5 represented a broader particle size distribution (Fig. 5). In addition, the crystalline structures were different for Sp10 and Sp5. Our analysis above (Fig. 9 and Table S3†) shows that the Sp5 sample was composed of the anatase phase, while the Sp10 sample consisted of about 15% rutile phase. In addition, the XPS analysis showed a slightly higher amount of Ti^3+^ and defects/oxygen vacancies on the surface of the Sp5 sample. It has been reported that the presence of Ti^3+^ on the surface of the anatase phase might enhance the activity as it provides additional active sites.^71^ In addition, anatase is known to experience a slower rate of electron–hole recombination, and thus better access to the catalytically active sites.^72^ Another indication of different characteristics of structural disorders between Sp10 and Sp5 is a slight difference in the grades of the samples' grey color^73^ observed after the spark-ablation and calcination. By expanding the range of available light wavelengths as well as by altering the crystalline phase, colored TiO_2_ can improve the efficiency and versatility of photocatalytic reactions compared to white TiO_2_ as shown in the present and previous studies.^73–75^

Both ZnO samples were composed of a typical wurtzite structure, but the Sp sample provided slightly better overall photocatalytic performance compared to the commercial ZnO (Fig. 12c) thanks to a higher amount of surface defects (41% of non-lattice oxygen compared to 37% on the commercial sample) and smaller particle size.

In general, the size of nanoparticles is considered a key factor for improving (photo)catalysis. When the particle size drops below 10 nm, the number of atoms that reach the surface increases rapidly, resulting in a high surface-to-volume ratio, *i.e.* high amount of available reactive sites, increasing the activity towards pollutant adsorption and photocatalytic oxidation.^76^ In addition, the decrease in particle size should increase photonic efficiencies because of the improved interfacial charge-carrier transfer rates. However, reducing the particle size below a specific threshold causes surface recombination processes to become dominant. This is due to the majority of electrons and holes being generated close to the surface, and surface recombination occurs faster than interfacial charge-carrier transfer processes. Therefore, an optimum particle size results in a maximal photocatalytic efficiency.^77^

#### Photocatalytic stability

Apart from the photocatalytic activity, the ability of a photocatalyst to repeatedly withstand the reaction conditions is another important aspect for the industrial viability of photocatalytic processes.^78^ Nevertheless, the conventional TiO_2_ and ZnO photocatalysts are known to suffer from the poisoning of active sites during the photocatalytic process due to the adsorption of low volatile intermediates and the complete avoidance of catalyst deactivation is not possible.^79,80^ For example, the deactivation of almost 37% and 55% in the second and third cycles, respectively, has been reported during toluene degradation on the benchmarked TiO_2_ photocatalyst.^81^ In our study, the photocatalytic stability was evaluated in four consecutive identical photocatalytic tests using the same sample (Fig. S12†). Both MnO_*x*_ and TiO_2_ Sp samples demonstrated superior performance stability with a minor reduction of degradation efficiency after four runs as compared to the sol–gel MnO_2_ and commercial TiO_2_ (Fig. 12d and e). In addition, both Sp materials displayed removal efficiencies of more than 90% after 30 min of reaction and up to 100% after 60 min (Table S7†). For MnO_*x*_, the removal efficiency remained 100% over the first three cycles and dropped by 2% during the fourth cycle. Similarly, the removal efficiencies were 100%, 99%, 99%, and 97% over the four cycles for TiO_2_. In contrast, the sol–gel prepared MnO_2_ and commercial TiO_2_ materials showed a significant reduction of photocatalytic degradation efficiencies compared to the first runs (Fig. 12d and e). The sol–gel MnO_2_ efficiencies dropped from 98% to 91%, 71%, and down to 65% over the four cycles. In the case of TiO_2_, both samples exhibited a noticeable yellowing of the irradiated area during the stability assessment (Fig. S13†). The observed yellowing of the irradiated area was attributed to the accumulation of intermediates on the surface of photocatalysts^82^ and pointed to the differences in the photocatalytic activity between the two samples. The color change was significantly more pronounced in the commercial TiO_2_ samples whereas just a slight hue of yellow was observed on the surface of Sp TiO_2_. This demonstrated a lower susceptibility of spark-ablated TiO_2_ to intermediate accumulation and opens avenues for further investigation into the underlying factors influencing the photocatalytic behavior and stability of Sp TiO_2_ samples. For commercial TiO_2_, the efficiency reduction was even worse as it decreased from 97% to 81%, 56% in the second and third cycles, and even down to 37% in the fourth cycle. In contrast, a marginal improvement was observed for the ZnO Sp sample compared to the commercial ZnO nanoparticles as the removal efficiencies were reduced significantly after the first cycle for both samples (Fig. 12f). Their removal efficiencies decreased from 60% and 55% in the first cycle to 20% and 17% in the fourth cycle for the Sp sample and commercial ZnO, respectively. The worse photocatalytic activity and stability of ZnO samples could be attributed to the significantly larger primary particle size of ZnO Sp and commercial ZnO (33 nm and 40–100 nm, respectively), which resulted in smaller specific surface area, *i.e.* reduced amount of active sites, and thus the poisoning effect is more pronounced. Even though ZnO Sp nanoparticles showed marginal improvement of both photocatalytic activity and stability, our findings demonstrate that the spark-ablation method has large potential for the production of photocatalytic nanoparticles with sustained durability as indicated by MnO_*x*_ Sp5 and TiO_2_ Sp5 performances. Similar to our study, Liu *et al.*^73^ synthesized grey rutile TiO_2_ using the sol–gel method with two-step calcination. For their grey TiO_2_ sample, enhanced chemical stability was observed over six cycles owing to the special nanostructure and the presence of interior oxygen vacancies.^73^ The special nanostructure played an important role also in the study of Zhao *et al.*,^10^ where the Mn_3_O_4_–MnO_2_ composite prepared by the conventional sol–gel method showed outstanding photocatalytic stability over five cycles. The abovementioned special nanostructure provided a strong self-regeneration capacity involving oxygen vacancies that were replenished by oxygen from the air.^10^

From the presented findings, it is evident that the spark ablation method can compete with the conventional catalyst synthesis methods in the production of catalytic materials with enhanced reactivity; what's more, it additionally offers simplicity and versatility in nanomaterial production without the usage of expensive precursors and reactants preventing impurities and waste generation. However, the energy demanding calcination step remains necessary for the production of efficient photocatalysts. In future studies, this additional step may be eliminated as the oxidation state could be simply tuned through the introduction of oxygen into the carrier gas during the spark ablation and deposition process. Follow-up studies could focus on the effect of different carrier gases on the properties and photocatalytic performance of spark ablated nanoparticles, similar to a recently reported study producing Co–Ni bimetallic nanoparticles.^83^ Furthermore, ensuring the mechanical stability of spark-ablated nanostructures on substrates poses a notable challenge for the practical implementation of the presented method, which requires further optimization.

## Conclusions

To the best of our knowledge, spark ablation has not been previously reported or evaluated for the production of photocatalytic nanoparticles. Nevertheless, it holds significant potential for advancing the current state by enabling environmentally friendly nanoparticle synthesis with distinct properties that could prove beneficial for photocatalytic reactions. In order to test this hypothesis and provide a comprehensive solution, our focus encompassed the complete trajectory: from the methodology behind nanoparticle synthesis, their strategic deposition, to an analysis of their inherent attributes and subsequent performances. In particular, spark ablation with direct immobilization of the produced nanoparticles onto a glass fibre substrate is introduced as a method for the production of functionalized filters for airborne organic pollutant removal. The filters loaded with MnO_*x*_, TiO_2_, and ZnO nanoparticles were calcined to improve the oxidation state and crystallization. Their photocatalytic activity and stability were evaluated under visible and UV irradiation. It is important to point out that the spark ablation procedure played a pivotal role in the enhanced performance when compared to the benchmark photocatalyst. Given the original nature of our study in terms of applying this technique to photocatalysis, an initial establishment was imperative using benchmark materials such as TiO_2_. The spark-ablated and calcined MnO_*x*_ and TiO_2_ samples demonstrated almost two times better photocatalytic activity towards toluene degradation along with sustained performance durability as compared to sol–gel MnO_2_ and commercial TiO_2_. The enhanced photocatalytic performance and outstanding durability are attributed mainly to (i) the small particle size (sub-20 nm particle size) and open nanostructure of particle deposition, *i.e.* high surface-to-volume ratio and pollutant and light accessibility, (ii) the presence of surface defects/oxygen vacancies, and (iii) the elimination of surface contamination during the production of nanoparticles. This facilitates a comprehensive comparison, affording a clearer picture of the merits and limitations of the spark ablation process in the context of photocatalytic applications. We show that the spark-ablation method is viable for air cleaning control applications. This method provides a simple, versatile and flexible, precursor-free and waste-free alternative to the traditional methods, representing an environment-friendly and green chemistry route for catalyst production. As a proof of concept, this study focused on the production of conventional photocatalytic materials, but spark ablation can provide viable production routes for a wide range of catalytic materials and their mixtures, especially if we consider the unlimited possibilities of doping and mixing for optimizing the process. Therefore, the technique introduced here has the potential of producing and optimizing new photocatalytic materials with unique properties.

## Data availability statement

All data are included in the manuscript or ESI;† further inquiries are available from the corresponding authors.

## Author contributions

S. Dr., S. Di, A. S.-O., and J. W. conceived and designed the experiments. S. Di. and A. S.-O. provided the spark generator and electrodes. Z. Z. did preliminary Sp tests and prepared ZnO Sp samples. S. Dr. prepared the photocatalytic filters functionalized with MnO_*x*_ and TiO_2_ using the spark generator and sol–gel methods. S. Dr. prepared and characterized the samples with XRD and SEM. S. Dr. performed the photocatalytic degradation and stability tests. M. G. performed Raman spectroscopy analysis. R. P. performed HRTEM and SAED imaging. O. S. analyzed the samples with XPS and helped with data treatment and interpretation. S. Dr. performed data treatment and representation, prepared graphs and figures, and wrote the manuscript with the support of J. W. and A. S.-O.

## Conflicts of interest

The authors declare that the research was conducted in the absence of any commercial or financial relationships that could be construed as a potential conflict of interest.

## Supplementary Material

EN-011-D3EN00348E-s001
