# Supplementary material for: Precursor- and waste-free synthesis of spark-ablated nanoparticles with enhanced photocatalytic activity and stability towards airborne organic pollutant degradation
Source: Environ Sci Nano. 2024 Jan 3;11(3):1023–43. doi: 10.1039/d3en00348e (PMC10939172; doi:10.1039/d3en00348e)
Supplement: EN-011-D3EN00348E-s001 [file EN-011-D3EN00348E-s001.pdf]

Electronic Supplementary Material (ESI) for Environmental Science: Nano.
This journal is © The Royal Society of Chemistry 2024

Supplementary material

Precursor- and waste-free synthesis of spark-ablated nanoparticles with enhanced photocatalytic activity and stability towards airborne organic pollutant degradation

Sarka Drdova[1,2], Gao Min[1,2], Olga Sambalova[2], Robin Pauer[3], Zhouping Zhou[4], Sofia Dimitriadou[5], Andreas Schmidt-Ott[4,5] and Jing Wang[1,2]

[1] Institute of Environmental Engineering, ETH Zurich, 8093 Zürich, Switzerland

[2] Laboratory for Advanced Analytical Technologies, Empa – Swiss Federal Laboratories for Materials Science and Technology, 8600 Dübendorf, Switzerland

[3] Electron Microscopy Center, Empa – Swiss Federal Laboratories for Materials Science and Technology, 8600 Dübendorf, Switzerland

[4] Chemical Engineering Department, Delft University of Technology, 2600 AA Delft, the Netherlands

[5] VSPARTICLE B.V., 2629 JD Delft, the Netherlands

Keywords: spark-ablation, precursor and waste-free nanoparticle production, photocatalysis, VOC degradation, manganese oxide, titanium dioxide, zinc oxide

Corresponding author: jing.wang@ifu.baug.ethz.ch

Supplementary tables

Table S1 Estimation of MnO_x Sp5 crystalline phase using Miller indices and calculated *d*-spacing

Crystalline phase	***Crystal Plane Miller indices***	***Referenced d-spacing***	***Measured d-spacing***
Ramsdellite	(110)	4.02	3.93
Pyrolusite	(110)	3.12	3.15
Hausmannite	(112)	3.08	3.02
Hausmannite	(200)	2.88	2.90
Hausmannite	(103)	2.76	2.69
Ramsdellite	(310)	2.55	2.54
Ramsdellite	(111)	2.32	2.34
Pyrolusite	(210)	1.97	1.99
Ramsdellite	(311)	1.90	1.88
Hausmannite	(105)	1.80	1.77
Ramsdellite	(221)	1.64	1.64
Hausmannite	(400)	1.44	1.43
Pyrolusite	(301)	1.31	1.32

Table S2 Estimation of TiO_2 Sp5 crystalline phase using Miller indices and calculated *d*-spacing

Crystalline phase	***Crystal Plane Miller indices***	***Referenced d-spacing***	***Measured d-spacing***
Anatase	(101)	3.53	3.69
Anatase	(103)	2.43	2.65
Anatase	(004)	2.38	2.18
Anatase	(200)	1.89	1.86
Anatase	(105)	1.7	1.64

Table S3 Size of nanoparticles obtained using XRD and TEM method and anatase content estimation

Sample	*D_{XRD} (nm)*	*D_{TEM} (nm)*	*Anatase XRD (%)*	*Anatase Bandgap (%)*
TiO2 Sp10	9.5	8.7	86%	82%
TiO2 Sp5	8.4	9.2	100%	98%
TiO2 P25	22		60%	68%

Table S4 Estimation of AOS and surface defects from XPS analysis for manganese oxide samples

Sample	*p(MnO_2 fit)*	*p(MnOOH fit)*	*AOS*[1]	*Lattice oxygen*[2]	*Non-lattice oxygen*[2]	*Adsorbed water*[2]	***Surface defects or OH groups***[3]
MnO_x 10 Sp	4.7%	95%	**3.05**	28814	24287	1765	**52%**
MnO_x 5 Sp	6.2%	94%	**3.06**	26294	28670	243	**46%**
MnO_x 1 Sp	6.7%	93%	**3.06**	27555	24279	512	**46%**
MnO_2	23.2%	77%	**3.23**	24823	22233	1315	**44%**

[1]Averaged oxidation state determined as (p(MnO_2 fit)·(4+)+p(MnOOH fit)·(3+)). [2]XPS peak area. [3]Ratio of surface defects or/and adsorbed OH groups

Table S5 Estimation of AOS and surface defects from XPS analysis for titanium dioxide and zinc oxide samples

Sample	*p(Ti(III) fit)*	*p(Ti(IV) fit)*	*AOS*[1]	*Lattice oxygen*[2]	*Non-lattice oxygen*[2]	*Adsorbed water*[2]	***Surface defects or OH groups***[3]
TiO_2 10 Sp	6.3%	94%	**3.82**	45007	19912	0	**31%**
TiO_2 5 Sp	6.4%	94%	**3.81**	42378	17719	0	**30%**
TiO_2 P25	8.9%	91%	**3.78**	34505	24856	85	**42%**
ZnO 10 Sp	-	-	**-**	27258	19035	0	**41%**
ZnO NanoArc	-	-	**-**	28801	17193	881	**37%**

[1]Averaged oxidation state determined as (p(Ti(IV)) · (4+) + p(Ti(III)) · (3+)). [2]XPS peak area. [3]Ratio of surface defects or/and adsorbed OH groups

Table S6 Nanoparticle mass loading on filter substrates, degradation rate constant k and mass-normalized degradation rate constant k_{norm}

Sample	***Mass loading g/m²****	***k (min⁻¹)***	***k_norm (min⁻¹)***
MnO_x Sp10	8.3	0.109	0.132
MnO_x Sp5	9.1	0.095	0.104
MnO_x Sp1	12.3	0.126	0.102
MnO_2 Nanoflakes	9.6	0.068	0.072
TiO_2 Sp10	6.4	0.045	0.047
TiO_2 Sp5	6.8	0.098	0.147
TiO_2 P25	6.8	0.068	0.100
ZnO Sp10	10.6	0.018	0.017
ZnO NanoArc	10.6	0.015	0.014

*The mass loading was determined by the mass of a filter before and after the deposition process per unit area of the filter.

Table S7 Toluene removal efficiencies after 60 min of irradiation

	MnOx Sp	***MnO_2***	***TiO_2 Sp***	***TiO_2 P25***	***ZnO Sp10***	***ZnO NanoArc***
Run1	99.7%	98.2%	99.6%	96.6%	60.3%	55.2%
Run2	99.7%	90.6%	99.4%	81.3%	34.0%	30.7%
Run3	99.6%	71.4%	98.9%	56.5%	27.8%	22.2%
Run4	99.4%	65.1%	97.0%	37.0%	20.2%	17.3%

Supplementary figures

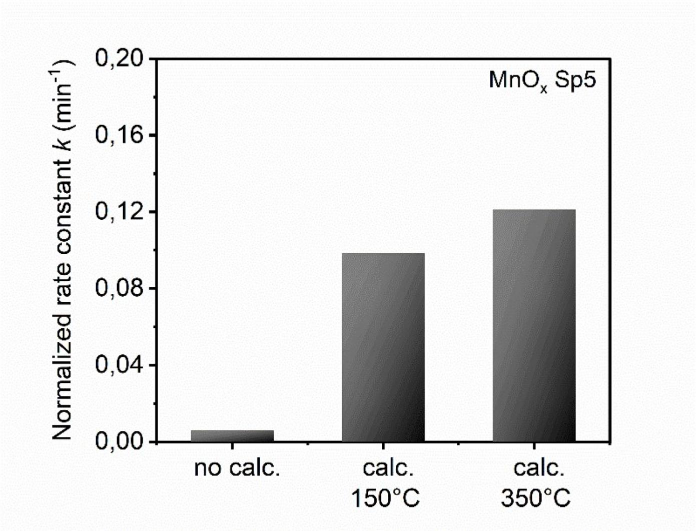

Figure S1 Preliminary experiments showing toluene degradation rate constant of MnO_x samples as-prepared with no calcination and calcined at 150 °C and 350 °C.

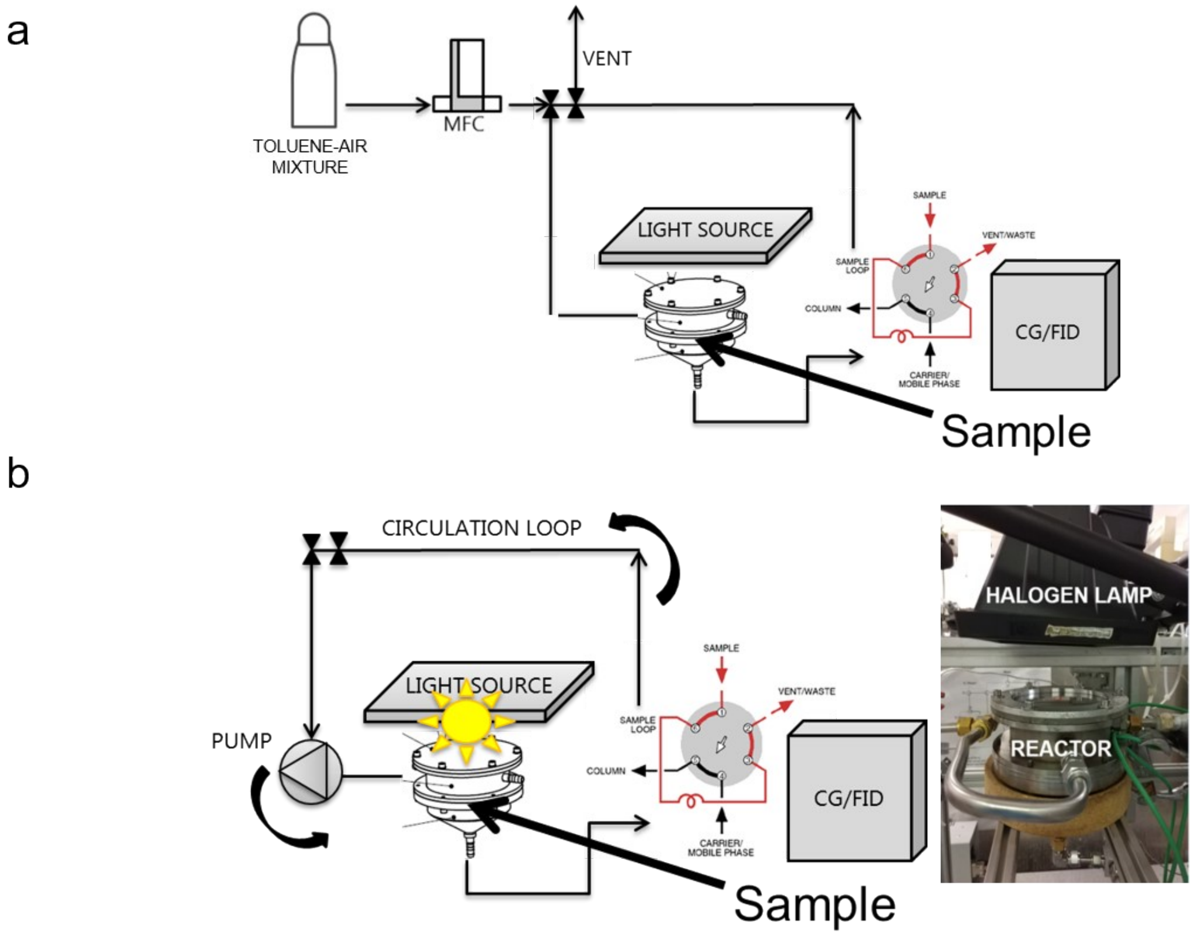

Figure S2 Schematic representation of photocatalytic degradation setup. a) configuration of toluene introduction into the reaction system and b) phase of toluene circulation (lamp off) and circulation and reaction (lamp on). The irradiation was facilitated by using either halogen lamp emitting UV-Vis-NIR simulating solar irradiation for manganese oxide materials or black light lamp emitting an UVA spectrum for titanium dioxide and zinc oxide materials. During the use of UVA irradiation, proper precautions were implemented to ensure the safety of occupants. These measures included adequately covering the experimental setup and wearing essential protective gear, such as lab coats, gloves, and UV-absorbing goggles or face shields. These precautions were implemented to minimize the risk of skin and eye exposure.

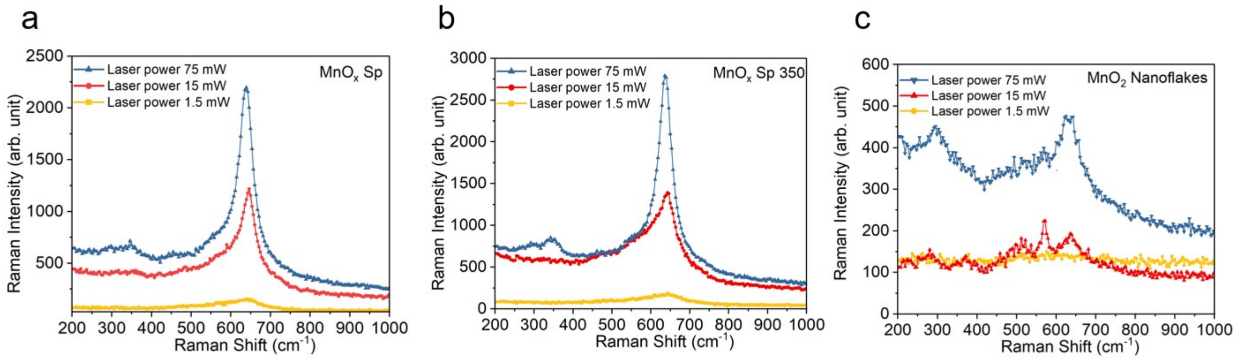

Figure S3 Raman spectra of spark-ablated a) as-prepared and b) calcined at 350 °C, and c) sol-gel prepared manganese oxide samples using distinct power conditions of 0.1%, 1%, 5%, and 10% of the full laser power (10% caused burning of samples, thus, data are not provided). A laser power level of 1% (equivalent to 15 mW) that was employed in conjunction with a 100x magnification and an accumulation time of 60 seconds offered a minimal degree of sample degradation.

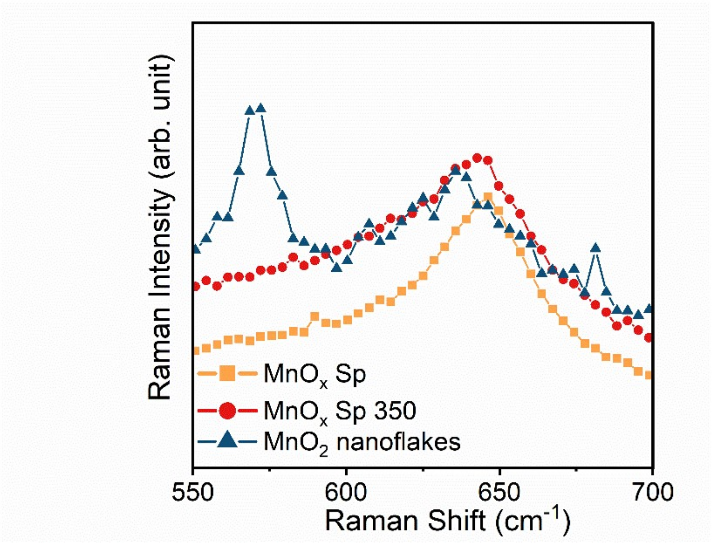

Figure S4 Comparison of Raman spectra at Mn-O vibration modes from 550 cm^{-1} to 700 cm^{-1} for spark-ablated a) as-prepared (MnO_x Sp) and b) calcined at 350 °C (MnO_x Sp 350), and c) wet-chemistry prepared (MnO_2 nanoflakes) manganese oxide samples.

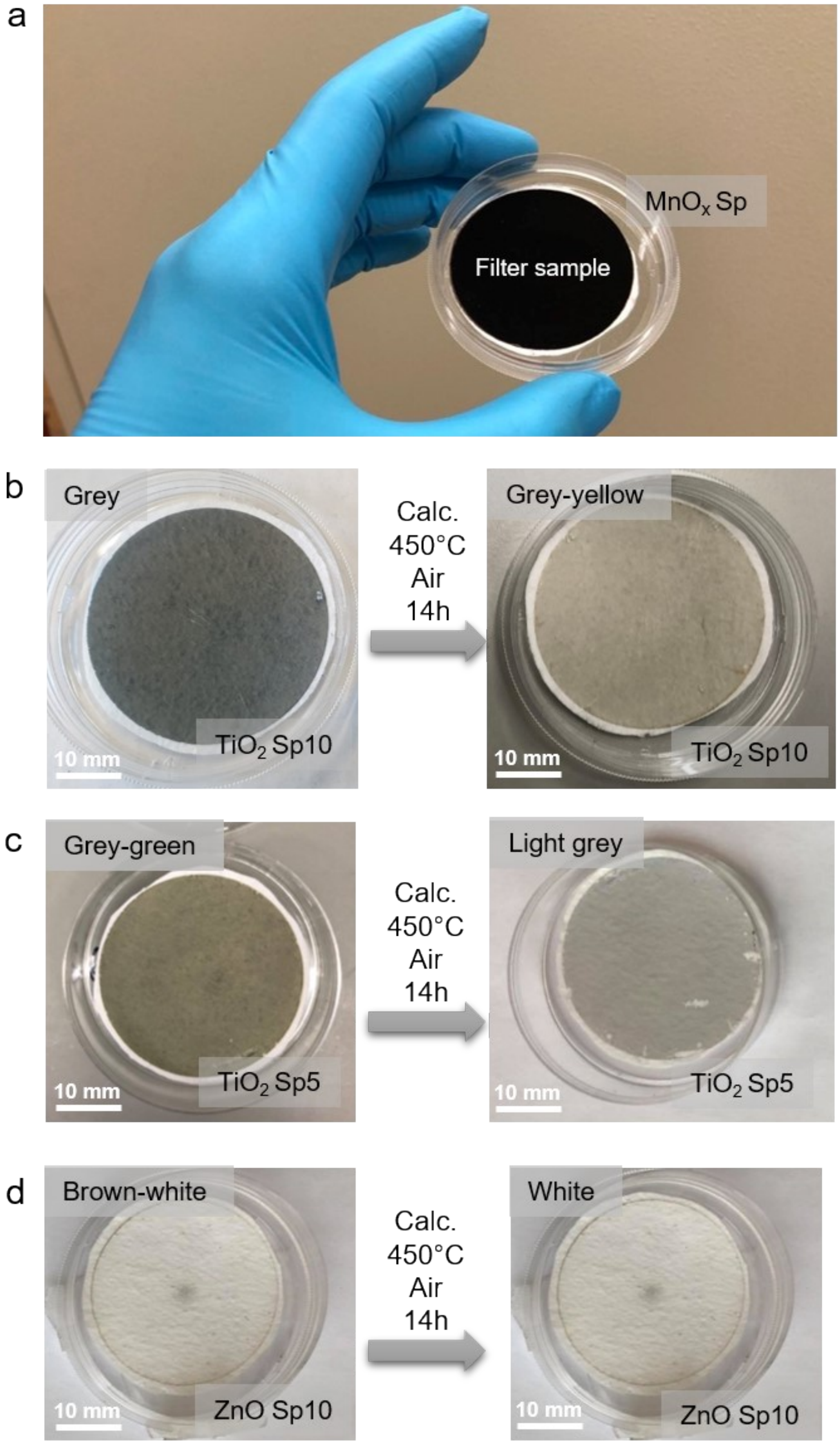

Figure S5 Photographs of spark-ablated samples for a) MnOx Sp representative sample, b) TiO_2 Sp10, c) TiO_2 Sp5, d) ZnO Sp10 before and after calcination.

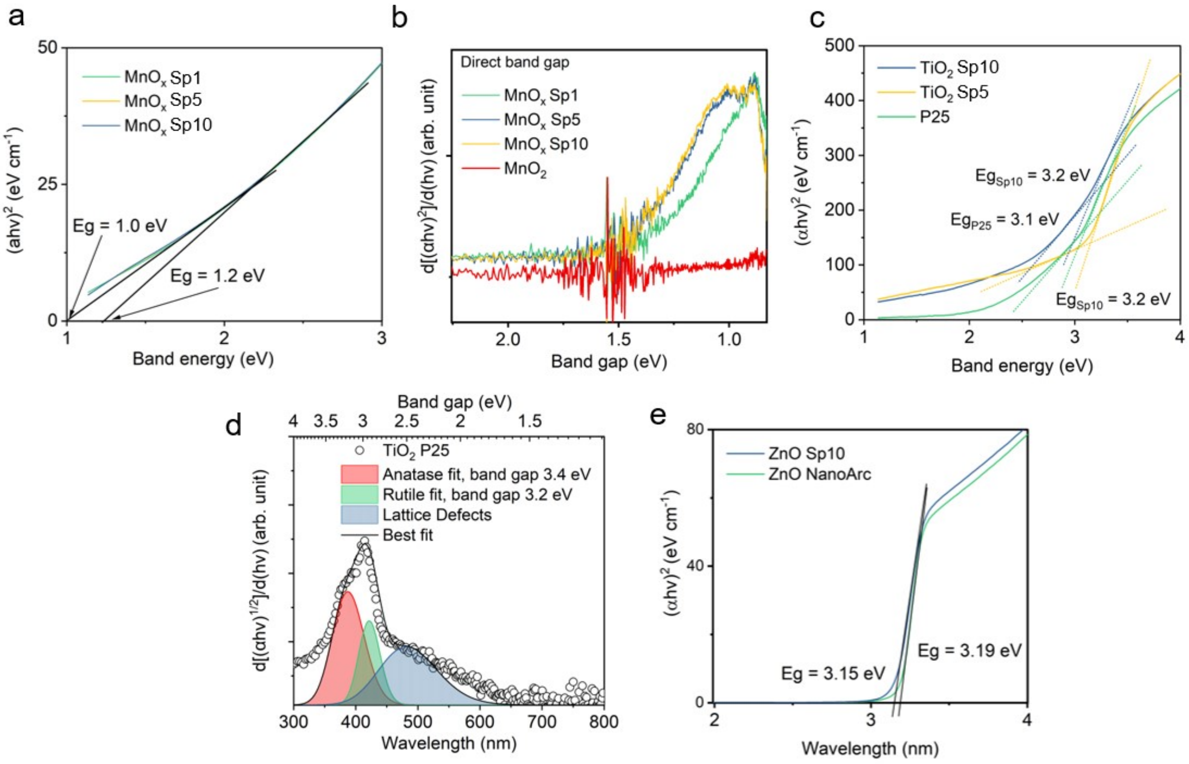

Figure S6 Tauc plot of band gap for a) MnO_x, c) TiO_2, and e) ZnO samples with indirect transition energies. Band gaps E_g were obtained from a), e) the extrapolation to $(\alpha h\nu)^2 = 0$, and from c) the intersection of extrapolation to $(\alpha h\nu)^2 = 0$ and an abscissa as the slope below the fundamental absorption, which is considered as the baseline in the sub-bandgap region of the Tauc plot. The 1st derivative of the Tauc plot b) and d) as a more sensitive method for the determination of band gaps.

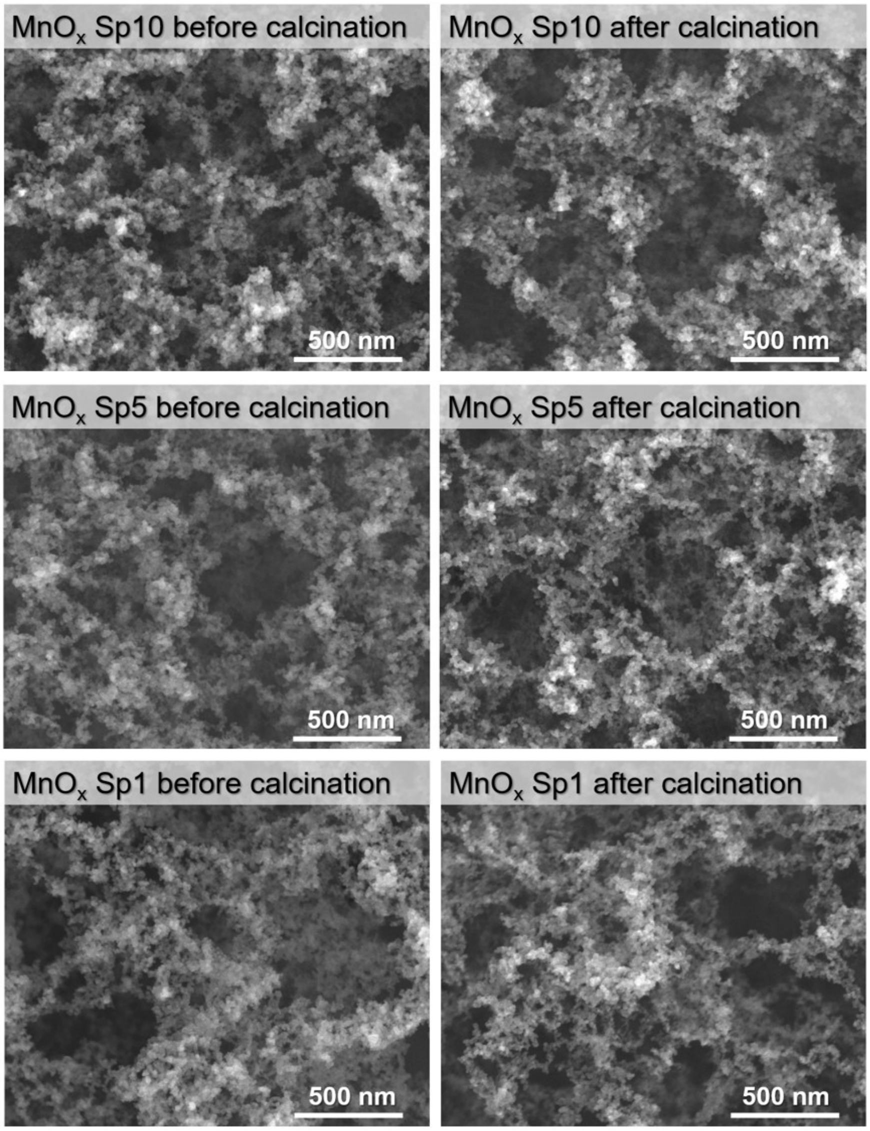

Figure S7 SEM images of MnO_x Sp samples before and after calcination.

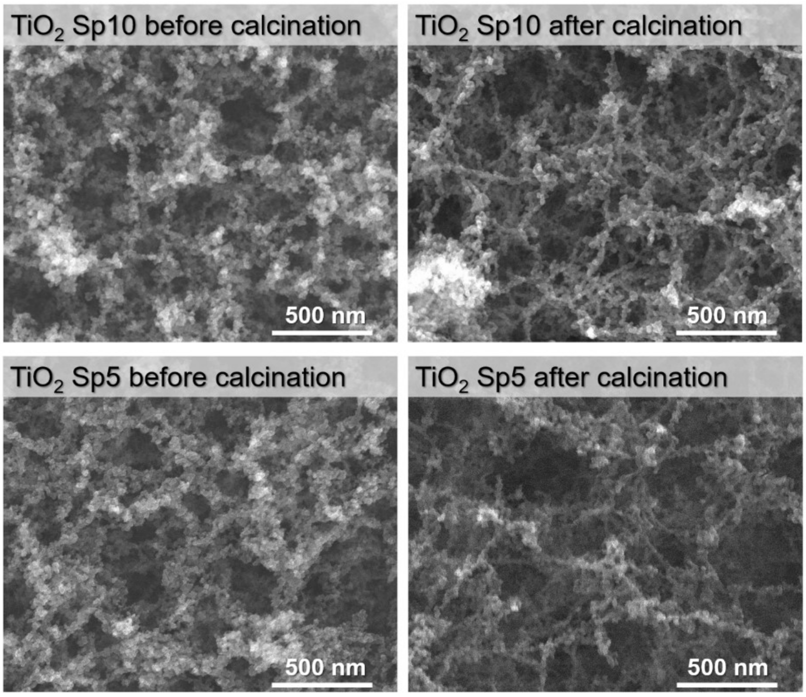

Figure S8 SEM images of TiO_2 Sp samples before and after calcination.

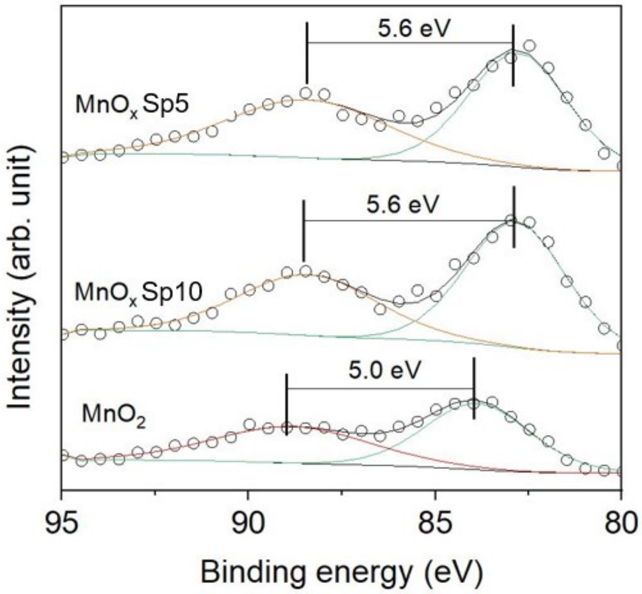

Figure S9 XPS spectra of Mn 3s core level splitting for MnO_x Sp5, MnO_x Sp10, and sol-gel MnO_2.

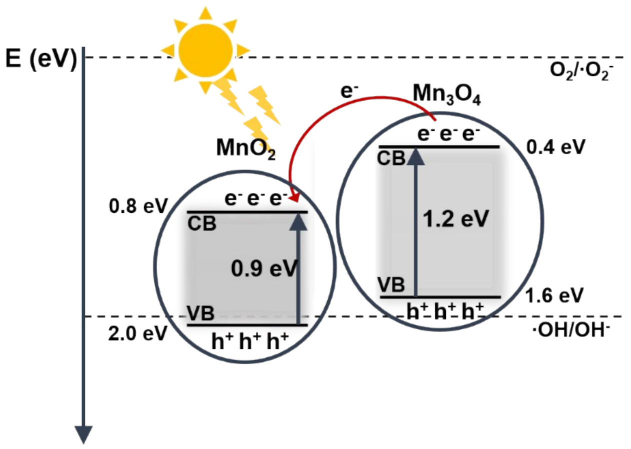

Figure S10 Suggested schematic representation of light induced charge carriers' generation and transfer for Mn_3O_4 and MnO_2 showing calculated values of valence band (VB) and conduction band (CB) determined from theoretical values of electron affinity and ionization energy and experimentally determined band gap energy.

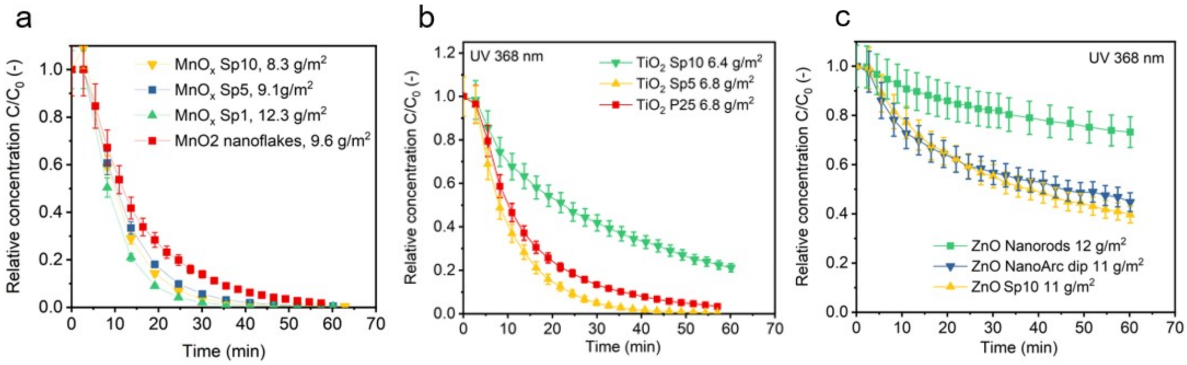

Figure S11 a) Relative concentration of toluene over the degradation period under UV-Vis-NIR irradiation for spark-ablated manganese oxide (MnO_x) nanoparticles and MnO_2 nanoflakes and under UV irradiation for spark-ablated c) titanium dioxide (TiO_2) and e) zinc oxide (ZnO) nanoparticles.

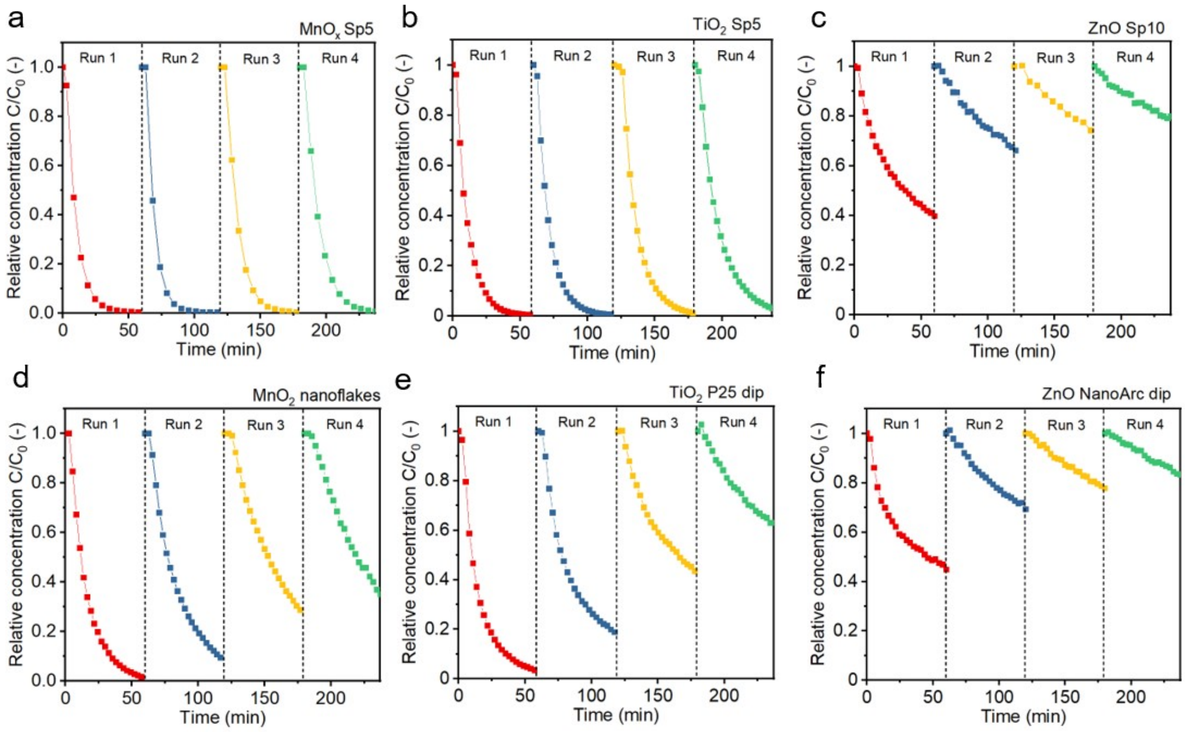

Figure S12 Photocatalyst stability over four consecutive toluene degradation cycles represented as relative concetration of toluene over irradiation time for a), spark-ablated and d) sol-gel manganese oxide (MnO_x and MnO_2 nanoflakes), b) spark-ablated and e) commercial titanium dioxide (TiO_2 Sp5 and TiO_2 P25), and c) spark-ablated and f) commercial zinc oxide (ZnO Sp10 and ZnO NanoArc) nanoparticles.

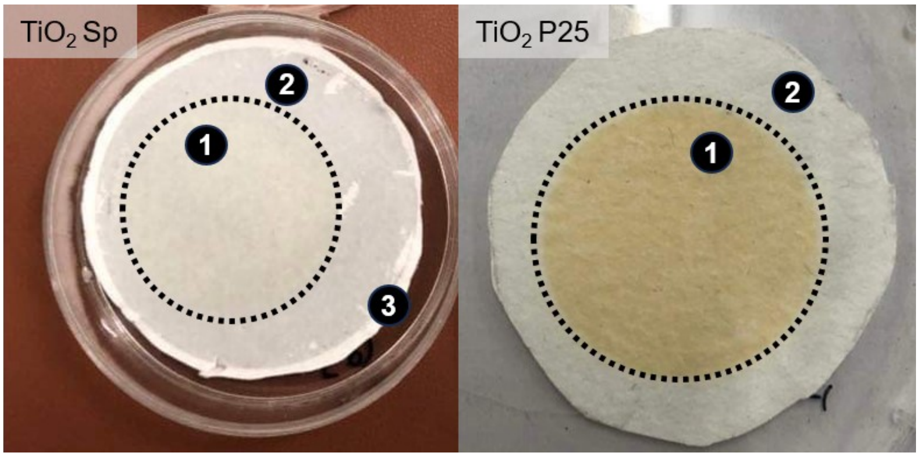

Figure S13 Photographs of filters with TiO_2 nanoparticles, obtained using the spark ablation method (left), and dip-coating of commercial TiO_2 nanoparticles taken after a photocatalytic stability assessment (four toluene degradation cycles). (1) shows the irradiated area with noticeable yellowing, which originated from fouling, i.e., the adsorption of intermediates. (2) shows the non-irradiated area where the nanoparticles maintained their original color, i.e., no adsorption of intermediates. (3) shows a non-coated and non-irradiated part of filter (blank). It is apparent that the fouling effect predominated on the surface of commercial TiO_2.
